# Application of Gelatin/Chitosan Films Loaded with Seaweed Extract of Ready-to-Eat Sea Cucumbers Preservation During Ambient-Temperature Storage

**DOI:** 10.3390/foods15183246

**Published:** 2026-09-14

**Authors:** Yingying Zhou, Yumeng Wei, Jingyi Huo, Lijuan Xu, Meng Li, Soottawat Benjakul, Xinru Fan

**Affiliations:** 1 Dalian Xinghai Bay Laboratory, Dalian 116023, China; 2 College of Food Science and Engineering, Dalian Ocean University, Dalian 116023, China; 3 International Center of Excellence in Seafood Science and Innovation, Faculty of Agro-Industry, Prince of Songkla University, Hat Yai 90110, Thailand

**Keywords:** brown algae extract, active packaging film, Ready-to-Eat Sea Cucumber (RTE-SC), ambient temperature storage, freshness preservation

## Abstract

Achieving ambient temperature storage of ready-to-eat sea cucumber (RTE-SC) remains a critical scientific challenge that urgently needs to be addressed in the industry. Recently, bioactive packaging materials have been increasingly applied in the aquatic product preservation, demonstrating their effectiveness in enhancing storage stability and extending the shelf-life. In this study, Seaweed extract (SE) was employed as a functional and bioactive component to fabricate gelatin/chitosan composite films (G-C–SE), and the preservative efficacy of the prepared films was systematically evaluated on ready-to-eat sea cucumber (RTE-SC) under ambient temperature storage. The SE was prepared and characterized in terms of extraction yield, total phenolic content, and total flavonoid content, all of which confirmed its satisfactory in vitro antioxidant and antimicrobial activities. The experimental results demonstrated that extraction using 60% (*v*/*v*) ethanol solvent yielded the most favorable outcomes, exhibiting satisfactory antioxidant and antimicrobial activities. Subsequently, the characterization and other physicochemical properties revealed that the incorporation of SE effectively facilitated the hydrogen-bonding interactions and improved the mechanical strength, barrier performance, and thermal stability of composite film matrix. When applied to the preservation of RTE-SC, the G-C–SE films exhibited remarkable quality-regulating effects. After 5 days storage, compared with the control group (CON), the protection of G3.3:C0.2−0.5% film (containing 3.3 wt% gelatin, 0.2% wt% chitosan, and 0.5 wt% *L. japonica* extract) resulted in a 26.07% reduction in the TCA-soluble oligopeptides content, a 10.39-fold decrease in glycosaminoglycan release, and an 11.28-fold suppression of TBARS elevation. Additionally, this treatment effectively retarded the rise in TVB-N accumulation, delayed textural deterioration, and maintained superior visual appearance throughout the storage period. These findings demonstrate that the G-C–SE composite film, as an active packaging material, can significantly mitigate the quality degradation of RTE-SC during ambient temperature storage, thereby providing a promising technological strategy for the application of bioactive films in aquatic food preservation.

## 1. Introduction

Sea cucumbers, belonging to the class *Holothuroidea* within the phylum Echinodermata, are a diverse group of marine invertebrates. Globally, the family comprises approximately 1700 identified species, of which about 140 species are distributed along the coast of China [1]. In 2024, the national aquaculture output of sea cucumber (*Apostichopus japonicus*) reached 326,200 tons, which assuming an 11.69% increase compared with the last year [2]. In recent years, rising living standards have driven growing consumer demand for high-nutritional seafood products, with RTE-SC emerging as a prominent ingredient in various traditional Chinese cuisines across both northern and southern culinary traditions. The rapid expansion of the prepared food products range has gained substantial momentum, because of their convenience of preparation and extended shelf-life. Among this RTE-SC based prepared dishes have increasingly been recognized as a promising direction for value-added processing in the industry.

Nowadays, sea cucumber processing technology has developed maturely, with two critical commercial product categories: dried sea cucumber and frozen RTE-SC predominant formats [3]. However, due to the intrinsic biological characteristics of fresh sea cucumber, which exhibits a potent autolytic capacity, the raw material is highly susceptible to exogenous stimuli during harvesting, transportation, and processing [4]. Such stress can trigger the programmed activation of endogenous proteolytic enzymes, leading to progressive disintegration of the sea cucumber body wall (SCBW) and concomitant release of intracellular components, thereby severely compromising both processing efficiency and storage quality of sea cucumber products [5]. To mitigate the adverse effects of autolysis, fresh sea cucumbers are typically subjected to brief pre-cooking and blanching treatments immediately after post-harvesting, followed by subsequent cooking (such as boiling, steaming, and high-pressure processing) and rehydration processing to yield the final RTE-SC product [6]. Nevertheless, because of the seasonal fishing restrictions and current preservation technologies limitations of the sea cucumber raw material, RTE-SC products are still relied on the traditional frozen storage and cold-chain logistics. How to extend the shelf-life of RTE-SC at room temperature and how to efficiently maintain the quality stability during storage are urgent scientific issues that need to be addressed, in order to achieve high-quality sea cucumber transportation and alleviate the pressure on sea cucumber processing industry.

Bioactive packaging materials have emerged as a research focal point in food preservation. Previous studies have demonstrated that arginine-modified chitosan-polyvinyl alcohol edible composite films possess excellent antioxidant and synergistic antimicrobial properties. When applied to the preservation of RTE-SC during 4 °C storage, these films effectively inhibited the weight loss, lipid oxidation, and microbial spoilage, while maintaining the textural and sensory quality of the samples, thereby highlighting the considerable application potential of chitosan-based active packaging in aquatic product preservation [4]. Despite its favorable film-forming capacity, chitosan—as a typical polysaccharide—is inherently constrained by its hydrophilic nature, which results in poor moisture barrier performance and insufficient mechanical properties. To overcome these limitations, blending chitosan with proteins has been proposed as an effective strategy. Through the formation of ionic and covalent crosslinking interactions, a composite gel network can be established, thereby enhancing the desired functional attributes of the resulting films. Gelatin is capable of forming dense and transparent film structures; however, it also suffers from drawbacks such as inadequate water resistance and suboptimal mechanical strength. When chitosan and gelatin are combined, they can form an interpenetrating polymer network that not only retains the superior film-forming properties of gelatin and the antimicrobial activity of chitosan, but also effectively improves the overall mechanical performance and structural stability of the composite films. This synergistic combination provides an ideal matrix for the sustained release of bioactive compounds on the surface of ready-to-eat sea cucumber, thereby enabling extended preservation efficacy.

In recent years, natural products with biological functional activities (such as polyphenols and organic acids) have gradually attracted the attention of the food packaging industry sector [7,8]. Some plant-derived extracts rich in antioxidant and antibacterial properties are employed as active controlled-release components, which can participate in the formulation of active packaging materials to prolong the shelf-life and enhance the storage stability of food materials and processed products [5]. Brown algae represent a significant marine plant resource, harboring a rich array of bioactive compounds. In China, the cultivation of edible brown algae yields substantial production volumes, with raw materials being readily accessible and exhibiting satisfactory food safety profiles. These brown algae are abundant in functional constituents, including polyphenols, polysaccharides, and organic acids, which endow them with notable antioxidant and antimicrobial activities [7]. Previous study has demonstrated that polyphenolic extracts (main component: phlorotannin) from *Ascophyllum nodosum* can effectively restrain the thermal degradation and maintain its resilient texture of SCBW based on its potential antioxidant properties [8]. Among the edible brown algae cultivated in China, *Laminaria japonica* (commonly known as kelp) ranks foremost in annual production, with its output reaching one of the highest levels worldwide. This species is rich in bioactive constituents, including fucoidan, polyphenols, and iodine, which have been extensively utilized in the food and pharmaceutical sectors, demonstrating a relatively high degree of industrial application [9]. *Hizikia fusiformis*, belonging to the same order Fucales as *A. nodosum*, is similarly abundant in phenolic compounds and exhibits comparable metabolic profiles and protein-protective capacities to those of *A. nodosum* [10]. As such, brown algae serve as promising natural quality-enhancing agents for the development of green active packaging materials.

As a consequence, we hereby propose the scientific hypothesis that the incorporation of brown seaweed extract as a functional filler into the gelatin/chitosan film-forming system could enable the construction of a stable three-dimensional network through hydrogen bonding and hydrophobic interactions. This strategy is anticipated to confer favorable mechanical properties and barrier performance to the packaging films, while simultaneously enabling the phenolic constituents to scavenge oxidative free radicals generated during the ambient storage of RTE-SC and to inhibit surface microbial proliferation. Accordingly, this system may serve as an ideal alternative research platform for elucidating the preservative mechanisms mediated by brown seaweed extract in sea cucumber preservation. Subsequently, the relevant experimental work was carried out accordingly. In this study, a composite active packaging film loaded with SE was fabricated and characterized for evaluating its preservative efficacy of RTE-SC during ambient-temperature storage. The comprehensive measurements of textural properties, freshness parameters, and other relevant quality indicators execute to establish a robust methodology for quality assessment and regulatory control of RTE-SC. The ultimate goal is to extend the ambient-temperature shelf-life and enhance the storage stability of RTE-SC products. The findings are expected to offer valuable theoretical insights for facilitating high-quality transportation and alleviating the processing pressures currently faced by the sea cucumber industry.

## 2. Materials and Methods

### 2.1. Materials and Chemicals

Cultured dried kelp (*Laminaria japonica*, 2 kg) was purchased from Xiapu, Fujian province, and dried *Hizikia fusiformis* (5 kg) was bought from Dongtou, Zhejiang province. Ready-to-eat sea cucumbers (RTE-SC; weight = 19.52 ± 2.10 g, length = 6.95 ± 0.50 cm, *n* = 90) was afforded from local market (Dalian, Liaoning province, China). Chitosan (CAS No. 9012-76-4) with a viscosity range of 100–200 mPa·s was purchased from Shanghai Macklin Biochemical Technology Co., Ltd. (Shanghai, China). Fish gelatin (CAS No.9000-70-8) was acquired for Sigma-Aldrich (Shanghai) Trading Co., Ltd (China). Other chemical reagents were procured from Macklin (Shanghai, China) and Aladdin (Shanghai, China).

### 2.2. Preparation of Seaweed Extracts (SE)

*L. japonica* and *H. fusiformis* were subjected to the pretreatment described by [11] with slight modification. The dried samples were washed through tap water to remove salt and sand, and were then rinsed with distilled water. Subsequently, the tidy samples were dried overnight in a convection oven at 50 °C to reduce the moisture content below 10%. The re-dried samples were ground into powder, sieved (80 mesh), and stored in a desiccator. Then, the samples were extracted with 15-fold volumes of 60%, 70%, and 80% (*v*/*v*) ethanol, respectively, in an ice bath under ultrasonication at 600 W for 10 min, and subsequently stirred for 2 h. The mixture was subsequently collected with a centrifugation of 7500× *g* for 25 min (4 °C). The supernatant was collected and evaporated separately via an evaporator at 45 °C. The superfluous ethanol within concentrated samples was eliminated with a gentle flow of nitrogen. The final extracted samples were freeze-dried and stored at −40 °C freezer until use.

### 2.3. Evaluation of SE Physicochemical Properties

#### 2.3.1. Extraction Yield

The extraction rate of SE was defined as the ratio of the dry weight of the final extract to the dry weight of the brown algae powders used for extraction.

#### 2.3.2. Total Phenolic Content (TPC) and Total Flavonoid Content (TFC)

The TPC was determined according to the Amin, et al. [12]’s method, which was utilized the gallic acid (0–500 mg/L) as the standard substance via Folin–Ciocalteu’s reagent. The reaction was carried out at ambient temperature for 45 min, and the absorbance was subsequently recorded at 760 nm. The method of Ahmad, et al. [13] was adopted to compute the TFC by the Aluminum oxide colorimetric method through catechin as a criterion. The catechin (0–400 mg/L) was used as a criterion for standard curve. The absorbance was read at 510 nm.

#### 2.3.3. Antioxidant Activities

The Trolox equivalents were applied to estimate the antioxidant activities of DPPH and ABTS radicals scavenging activities, and ferric reducing antioxidant power (FRAP), which following the procedures of [13]. For DPPH radicals scavenging activity assay, 0.15 mol/L DPPH was mixed with an equal volume of the sample (1.5 mL) at room temperature for 30 min. The absorbance was detected at 517 nm. The ABTS radicals scavenging activity assay was performed by mixing 0.15 mL with 2.85 mL ABTS working solution. The absorbance was measured at 734 nm after incubation for 45 min. The 0.15 mL of sample was added to 2.85 mL ferric-TPTZ working solution for 30 min to obtain the absorbance at 593 nm.

#### 2.3.4. Antibacterial Activities

The minimum inhibitory concentration (MIC) and minimum bactericidal concentration (MBC) values of the brown algae extract were estimated via Mueller–Hinton broth microdilution, which was obtained the bacterial strains of *Escherichia coli* (*E. coli*, ATCC 25922) and *Staphylococcus aureus* (*S. aureus*, ATCC 6538). Briefly, the extract (200-1.56 mg/mL) was mixed with TSB medium (containing 10^6^ CFU/mL bacterial suspension) at a 1:1 ratio in 96-well microplate and incubated at 37 °C for 24 h [14].

### 2.4. LC-MS Identification of Compounds in SEs

The compounds in the brown algae extract were identified using an Agilent LC-QTOF/MS liquid chromatography mass spectrometer (129 InfinityII LC-6545Q-TOF, Agilent Technologies, Santa Clara, CA, USA). The equipment included an automatic sampler (G2179B), a binary pump (G7120A), a column chamber (G7116B), and a diode array detector (G7117A). The samples was separated by the Zorbax Eclipse Plus C18 column (150 mm × 2.1 mm, Agilent Technologies, Santa Clara, CA, USA) with an guard column (5 mm × 2.1 mm). Mobile phases A and B were 2% (*v*/*v)* acetic acid dissolved in water and Acetonitrile: Water: acetic acid (50:49.5:0.5, *v*/*v*/*v*), respectively. The flow rate was 0.2 mL/min, and the temperature was 25 °C. The gradient program was as follows: 95% A + 5% B constant (0–40 min), 100% B (40–45 min), 95% A + 5% B constant (45–50 min). The mass spectrometer acquisition method was to use dual AJS ESI as the ion source, ranging from 100 to 1700 ms (*m*/*z*). The scanning segment was performed in negative mode electron spray ionization. The results indicated that this method has good resolution and high sensitivity. The liquid chromatography/mass spectrometry was used for identification in the negative mode. To identify the components, the collected data was processed using the software “MassHunter METLIN metabolite PCD (Personal Compound)” and version 8 of PCDL (Personal Compound Database and Library). The reference mass was used to verify the resolution of the high mass spectrometry analysis. This confirmed that the dissociation fragments induced by the negative ion source can provide accurate mass information. The ESI chromatographic sub-section was evaluated using the lc/ms tool system (formula calculator) for the quality value (*m*/*z*). The selected peaks on the chromatogram and mass were determined by the system, while the mass was analyzed using ESI scanning to determine the charge ratio (*m*/*z*) of the selected peaks.

### 2.5. Preparation of Gelatin/Chitosan Films Loaded with Optimized Seaweed Extract (G-C–SE)

The preparation of gelatin and chitosan blend films loading with SE was followed the method of [15]. Briefly, a 6.2 wt%, 6.4 wt%, 6.6 wt% concentration of gelatin solution was prepared respectively with a 60 °C incubation for 2 h until complete solubilization, which was regard as the gelatin film forming solution (G). A 0.8 wt%, 0.6 wt%, 0.4 wt% chitosan was dissolved in 1% (*v*/*v*) acetic acid under stirring at room temperature overnight to make the chitosan film forming solution (C). The G/C blend was mixture at the ratio of 1:1 coming up to a total concentration of 3.5 wt%. Subsequently, 30% glycerol was separately added to the G/C solution with the addition of 0%, 0.5%, 1.0% (*w*/*w*) SE. The following nine sets of samples were obtained: G3.1:C0.4−0%, G3.1:C0.4−0.5%, G3.1:C0.4−1.0%; G3.2:C0.3−0%, G3.2:C0.3−0.5%, G3.2:C0.3−1.0%; G3.3:C0.2−0%, G3.3:C0.2−0.5%, G3.3:C0.2−1.0%. The 5 × 5 cm^2^ cell of silicon casings was devoted to formulate the film samples with an overnight air drying and an equalized drying (LC-HSP-70BE, Zhejiang Lichen Instrument Technology Co., Ltd., Shaoxing, China) at the relative humidity of 50 ± 5% for 48 h.

### 2.6. Characterization of G-C–SE Films Functional Characteristics

#### 2.6.1. Color and Light Transmittance

The color characteristics of film samples were appraised via a colorimeter (Hunterlab, Reston, VA, USA) following the measurement of Mutlu, N. [16]. The results were expressed as the brightness (L*), red-green value (a*), yellow-blue (b*), chroma (C*), hue (H*), and total color difference (*ΔE*). The white board was applied to the standard reference (L*_0_ = 92.82, a*_0_ = −1.24, b*_0_ = 0.50). The light transmission of the films was estimated from 200 nm to 800 nm by the UV-Vis spectrophotometer (UV-1800, Shimadzu, Kyoto, Japan) based on the method of [17].

#### 2.6.2. Evaluation of Thickness, Mechanical Properties, and Water Vapor Permeability (WVP)

The thickness of G-C–SE films was measured using a micrometer (32CHQF1030, Deqing Shengtaixin Electronic Technology Co., Ltd., Huzhou, China) with 10 individual film samples obtaining 5 random points. The mechanical properties of tensile strength (TS) and elongation at break (EAB) of the film were determined based on the previously reported method [18].

The WVP parameter was determined as previously described by Awal, et al. [19]. The film samples were placed on the aluminum penetration cup with dried silica and sealed securely. These cups were displayed in a constant temperature and humidity condition (25 ± 2 °C, 50 ± 5% relative humidity), and weighing interval 1 h for an accumulation of 10 h.

#### 2.6.3. Fourier Transform Infrared Spectroscopy (FTIR) Analysis

The FTIR spectra of the selected films were recorded with an ATR diamond cell (PIKE Technology Inc., Madison, WI, USA) assembled into Bruker Equinox 55 FTIR spectrometer (Bruker Corporation, Ettlingen, Germany). The normalization was performed before interpretation for all the obtained spectra [20].

#### 2.6.4. Scanning Electron Microscopy (SEM)

The surface and cross section morphology of the selected samples were visualized using the Quanta 400FEI scanning electron microscope (Eindhoven, The Netherlands).

#### 2.6.5. Thermogravimetric Analysis (TGA)

The thermal stability of the selected films was evaluated using a simultaneous thermal analysis instrument (STA-8000, PerkinElmer, Norwalk, CT, USA) with a heating rate of 10 °C/min from 30 to 800 °C, and a nitrogen flow rate of 20 mL/min. The thermal degradation temperature and weight loss of the selected films were determined for the samples [21].

### 2.7. Application of G-C–SE Films in RTE-SC Preservation

The rehydrated RTE-SC (n = 60, weight = 19.52 ± 2.10 g; length = 6.95 ± 0.50 cm) was purchased from a local fish market. The RTE-SC was wiped with sterile paper to remove the releasing water, and placed on a sterile petri dish. Different types of films were used for packaging: a commercial PVC film as the control group (CON), a G-C film with or without seaweed extract. These films needed to be exposed to ultraviolet light for 30 min before packaging. Subsequently, the packaged sea cucumber samples were stored at 25 °C for 5 days, and collected for destructive analysis.

#### 2.7.1. Textural Properties

The RTE-SC body wall was cut into small cubes (1 cm × 1 cm × 0.5 cm) and measured by texture analyzer (TMS-PRO, Food Technology Corporation, Sterling, VA, USA) equipping with P/25 probe. The texture profile analysis (TPA) mode with testing speed of 1.0 mm/s, strain of 50%, trigger force of 0.1 g was applied for the destructive test. Each group with six parallels [5].

#### 2.7.2. TCA-Soluble Oligopeptides and Glycosaminoglycans (GAGs)

The TCA-soluble oligopeptides results was followed the method of Du, et al. [22]. Briefly, 0.5 g of RTE-SC samples was homogenized with 2 vol of 0.02 mol/L PBS buffer (pH 7.4) with a centrifugation of 12,000× *g* for 15 min (4 °C). The supernatant was regarded as the water-soluble collagen solution, which reacted with equal vol of 20% TCA-solution placing in iced-water for 20 min. After that, the mixture was centrifuged at 13,500× *g* for 15 min (4 °C) testing with Folin reagent. The protein content was expressed as the TCA-soluble oligopeptides result. For the GAGs measurement, the diluted water-soluble collagen solution was reacted with 1,9-Dimethylmethylene blue solution at 525 nm [23].

#### 2.7.3. pH Value

The RTE-SC samples was homogenized with 10 vol of sterile distilled water, and filter with filter paper. The pH value was performed with the filtered solution [16].

#### 2.7.4. Total Volatile Basic Nitrogen (TVB-N), Thiobarbituric Acid (TBARS), and Total Viable Count (TVC)

The determination of TVB-N, TBARS content and TVC result was conducted with Chinese national standard GB 5009.228-2016 (Determination of Volatile Basic Nitrogen in Food), GB 5009.181-2016 (Determination of Malondialdehyde in Food), and GB 4789.2-2022 (Food Microbiology Examination, Determination of Colony Count), respectively [24,25,26].

### 2.8. Statistical Analysis

All the experiment data appeared as mean ± standard deviation in triplicate. Multivariable Statistic was conducted by SPSS 27.0 (SPSS Inc., Chicago, IL, USA) via one-way ANOVA, where *p* < 0.05 represented the difference between groups with significance. All diagrams were plotted getting through Origin 2021 (Origin-Lab, Northampton, MA, USA).

## 3. Result and Discussion

### 3.1. The SE Physicochemical Properties and Identification

#### 3.1.1. Effects of Different Ethanol Concentrations on the Physicochemical Properties of SE

The extraction yield, TPC, TBC, and antioxidant activities of all extracts obtained using different ethanol concentrations as the extraction solvent are presented in Table 1. The results indicated that ethanol concentration exerted a notable influence on both the polyphenol extraction efficiency and the corresponding antioxidant capacity. Compared with other ethanol concentrations, 60% ethanol yielded the highest polyphenol extraction rates, with values of 3.16 ± 0.15% (*L. japonica*) and 6.26 ± 0.17% (*H. fusiformis*) for the two seaweed species, representing increases of 13.67% and 17.45%, respectively, compared to those obtained with 70% ethanol. Hence, an appropriate ethanol concentration enhances the efficient extraction of phenolic compounds with profitable antioxidant properties. Excessively high ethanol concentrations favor the co-extraction of lipophilic impurities, which compete with polyphenols for solvent accessibility and binding sites, thereby diminishing polyphenol yield and purity. This result aligns with the cashew (*Anacardium occidentale* L.) leaves phenolic extraction [11]. Concurrently, the extracts obtained at 60% ethanol exhibited the maximum TPC and TFC, recorded as 16.40 ± 0.24 mg/g and 141.25 ± 4.51 mg/g, respectively. At the optimal ethanol concentration (60%), the antioxidant activities of both samples displayed considerable results, with those from *H. fusiformis* demonstrating significantly higher values than those from *L. japonica* (*p* < 0.05). Especially, the DPPH free radical scavenging activity, ABTS free radical scavenging activity, and iron ion reduction capacity of *H. fusiformis* were184.26 ± 0.33 μmol/g, 1133.01 ± 17.94 μmol/g, and 10.81 ± 0.22 μmol/g respectively. This demonstrates that the antioxidant capacity of *H. fusiformis* is significantly higher than that of *L. japonica* sample. This enhanced activity is plausibly attributable to the higher phenolic content, as the abundance of hydroxyl groups, determined by the number and positional arrangement on the aromatic rings, is directly associated with the radical-scavenging potential of polyphenolic compounds [27]. Meanwhile, the ABTS radical scavenging activity of the samples was significantly higher than their DPPH radical scavenging activity (*p* < 0.05), with a considerable disparity. This suggests that electron transfer pathways contribute more prominently to the antioxidant process of the bioactive components present in the extract. The ABTS^+^ radical maintains good stability in ethanol–water mixed media and, compared to DPPH, exhibits better compatibility with extract constituents of varying polarity, thereby enabling a more comprehensive reflection of the total electron-donating capacity of the sample. The FRAP value was determined to be 10.81 μmol/g, indicating a relatively low overall level, which implies that the extract possesses a weaker capacity to reduce Fe^3+^ compared to its ability to scavenge nitrogen-centered radicals. This phenomenon may be attributed to the absence of structural moieties with high Fe^3+^-reducing efficiency in the extract, and further suggests that its antioxidant activity is predominantly mediated through direct free radical quenching, while the metal ion reduction pathway plays a comparatively limited role in its overall oxidative inhibition mechanism [28].

The antimicrobial activities revealed that *H. fusiforme* extract exhibited the strongest inhibitory effect against the Gram-positive bacterium *S. aureus*, with the MIC value of 1.56 mg/mL and the MBC data of 6.25 mg/mL. These values were considerably lower than those reported for *L. japonica* extract (MIC = 6.25 mg/mL; MBC = 25 mg/mL), underscoring that *H. fusiforme* extract exhibits superior bacteriostatic and bactericidal efficacy against *S. aureus* extract. The MBC/MIC ratio serves as a useful parameter for distinguishing between bacteriostatic and bactericidal effects. Generally, a ratio of ≤4 is considered indicative of bactericidal activity. In the present study, the MBC/MIC ratios for both the *H. fusiforme* and *L. japonica* extracts were exactly 4, further confirming that their antimicrobial action against *S. aureus* is predominantly bactericidal rather than merely transiently inhibitory. However, the overall MIC and MBC values of the *L. japonica* extract were significantly higher than those of *H. fusiforme*, indicating that a substantially higher concentration of the former is required to achieve an equivalent bactericidal effect. In contrast, both SEs exhibited markedly diminished antimicrobial activities against the Gram-negative bacterium *E. coli*. For *H. fusiforme*, both MIC and MBC reached ≥50 mg/mL, whereas *L. japonica* showed an MIC of 25 mg/mL and an MBC of 50 mg/mL. This differential susceptibility is explained that the structural features in bacterial cell envelopes: the outer membrane of Gram-negative bacteria, rich in lipopolysaccharide, functions as a selective permeability barrier that impedes the cellular uptake of hydrophobic or amphiphilic bioactive compounds—particularly phenolic and flavonoids—present in the brown algal extracts [11]. *L. japonica* extract exhibited relatively better inhibitory activity against *E. coli* compared to its effect on Gram-positive strains, which may be attributable to compositional differences between the two seaweed species. It is plausible that certain polysaccharide fractions present in *L. japonica* are more prone to interact with the surface of Gram-negative bacterial cells. Nevertheless, this effect appears to be confined to bacteriostatic action, as the MBC remained at 50 mg/mL, indicating only limited bactericidal capacity. These results demonstrate that *H. fusiforme* possesses superior anti-staphylococcal potency and a more favorable bactericidal profile than *L. japonica*.

#### 3.1.2. Quantitative Analysis of SE Components

The qualitative analysis using LC-MS, coupled with spectral library matching, contributed to the tentative identification of 22 and 23 distinct compounds from *H. fusiforme* and *L. japonica* extracts, respectively. As shown in Tables S1 and S2, the major compounds encompassed a diverse array of chemical classes, including polyols, organic acids, nucleotides, amino acids, and various plant-derived secondary metabolites. A comparative analysis of compositional profiles, structural features, and proposed mechanism of action revealed that both SEs shared certain bioactive scaffolds (phenolic hydroxyl and carboxyl groups), which are established the contribution of antioxidant or antimicrobial activities.

Notably, vanillic acid 4-sulfate (*H. fusiforme*) and pyrocatechol (*L. japonica*) contain ortho-diphenolic or monophenolic hydroxyl groups capable of donating hydrogen atoms to stabilize DPPH and ABTS^+^ radicals, thereby conferring measurable radical-scavenging capacity. Nevertheless, the *H. fusiforme* extract demonstrated significantly antioxidant potency, which probably ascribe to the greater diversity of its bioactive constituents (itaconic acid, citric acid, secondary metabolites, and sphingolipids). In addition, the larger proportion of low molecular weight active compounds (such as vanillic acid 4-sulfate and itaconic acid) contribute to a higher water solubility, facilitating rapid diffusion into the internal matrix of high-moisture aquatic products, thereby enhancing their accessibility for oxidative reactions. Furthermore, phenolic acid derivatives bearing sulfonate or carboxyl groups are predicted to maintain conformational stability under low-temperature and high-salinity conditions typical of seafood storage and processing environments.

In contrast, the antioxidant capacity of *L. japonica* extracts relied on a narrower spectrum of phenolic compounds, primarily represented by omega-hydroxymoracin N and the naphthoquinone derivative myrsinone. Although both exhibit potent intrinsic radical-scavenging activity, their action is relatively confined to direct free radical neutralization, with limited efficacy in mitigating metal ion-catalyzed oxidative processes. Consequently, the overall antioxidant system of *L. japonica* is less multifunctional. Furthermore, the comparatively higher molecular weights of omega-hydroxymoracin N (326.12 Da) and myrsinone (294.18 Da) result in lower molar concentrations per unit mass than the low molecular weight organic acids prevalent in *H. fusiforme*. While uric acid (168.03 Da) was also detected as a minor constituent in *L. japonica*, its low abundance precluded a substantial contribution to the overall antioxidant effect.

### 3.2. Characterization of G-C–SE Film

#### 3.2.1. Appearance, Color Characteristics and Light Transmittance

**Figure 1:**
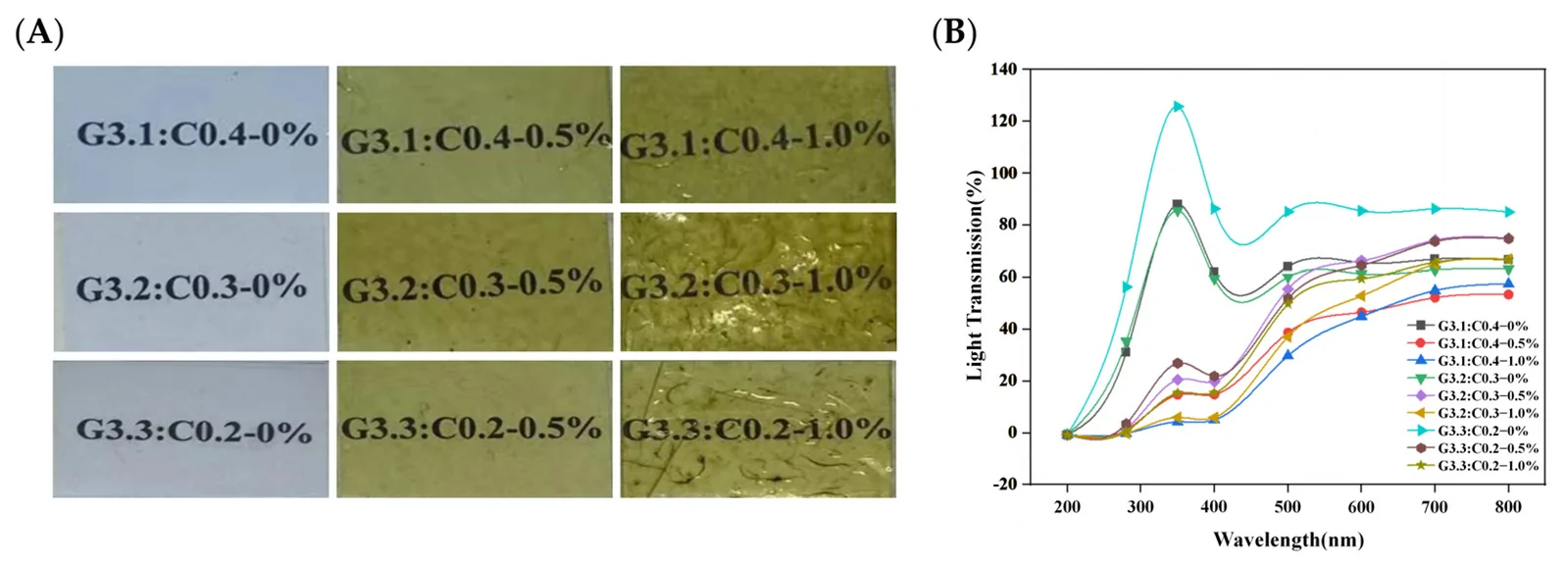
Visual appearance (**A**), the light transmittance (**B**) of G-C–SE films.

In Figure 1A, the visual appearance of the prepared films affected by the SE concentration. A progressive darkening greenish color was observed as the accumulation of SE content. Noteworthily, when the SE addition upped to 1.0%, distinct fibrous insoluble particulates became visible within the film matrix. The film formulations of G3.1:C0.4 and G3.2:C0.3 with the incorporation of SE led to a concentration dependent decline in L* values, and concomitant increase in b*, C*, and *ΔE*. In contrast, the a* and H* parameters exhibited a non-monotonic response, initially decreasing and subsequently rising (Table 2). These chromatic alterations collectively indicate that SE addition reduced surface lightness and conferred a yellowish-green hue to the films, resulting in substantial overall color deviation. Such changes are plausibly attributable to the intrinsic pigments (chlorophylls and carotenoids) present in SEs, combined with the pale-yellow coloration of the film polymer matrix. Moreover, the physical interactions between SE particles and the biopolymer network may promote localized phase segregation or pigment clustering, thereby amplifying color intensity and heterogeneity. In the case of G3.3:C0.2 films, distinct colorimetric trends were observed relative to the SE-free control. The a* values decreased progressively with increasing SE concentration, confirming a pronounced shift toward green dominance. The L* and H* values exhibited an initial decline followed by partial recovery as SE concentration increased, whereas the b*, C*, and *ΔE* values displayed an opposite pattern. These biphasic responses suggest that SE-induced color modulation is highly dependent on the specific polymer ratio and complex interactions among pigment components, matrix composition, and particle dispersion behavior [29].

Optical transparency is a critical parameter for edible film materials, as it directly influences consumer acceptance in food packaging applications. The light transmission through polymer-based films is intrinsically governed by their internal structural organization, wherein densely packed architectures typically impede the passage of visible light. Accordingly, the transparency of all prepared G-C–SE composite films exhibited a progressive decline with increasing SE concentration (Figure 1B). The film samples devoid of SE expressed the highest overall transparency, whereas those containing SE displayed nearly zero ultraviolet (UV) transmittance within the wavelength range of 200–290 nm, illustrating a marked enhancement in UV-blocking capability imparted by the SE. This pronounced photoprotective effect might attribute to two synergistic mechanisms: (1) the presence of aromatic compounds within SE proposes strong UV–vis absorption capabilities; (2) the increased SE content promotes a more homogeneous polysaccharide network derived from the seaweed matrix, thereby intensifying both light scattering and absorption. Additionally, intermolecular interactions among SE constituents, gelatin, and chitosan may induce conformational rearrangements within the film microstructure, further modulating the optical behavior [30]. Noteworthily, when the G-C mixture ratio was incrementally adjusted from 3.1:0.4 to 3.3:0.2, transmittance consistently elevated. The maximum transmittance was 125.67% in G3.3:C0.2−0% film, expressing that a higher gelatin proportion favors the formation of a more optically permissive matrix structure.

#### 3.2.2. Thickness, Water Vapor Permeability (WVP) and Mechanical Properties

As presented in Table 3, the thickness results of the fabricated G-C–SE films, measured at varying SE incorporation levels, fell within a narrow range of 0.023 to 0.029 mm. A slight incremental increase in film thickness was observed upon SE addition; however, the magnitude of this variation remained relatively modest, which might attribute to the low dosage levels of SE employed in the film formulations. The WVP of the G3.1:C0.4−0% film showed the lowest value of 5.91 × 10^−11^ g m^−1^ s^−1^ Pa^−1^. Nevertheless, a statistically significant elevation in WVP results was demonstrated via the increase of SE cooperation (*p* < 0.05), which assumed a highest results in G3.3:C0.2−1.0% sample of 7.65 × 10^−11^ g m^−1^ s^−1^ Pa^−1^. These results explained that the films devoid of SE exhibited superior barrier properties relative to their SE-supplemented counterparts. The observed deterioration in barrier performance may be rationalized by two plausible mechanisms. On one hand, the incorporation of SE could potentially disrupt the structural continuity of the polymer matrix, thereby facilitating the permeation of water vapor molecules. On the other hand, the formation of hydrogen-bonding interactions between SE and gelatin–chitosan matrix might reduce the overall hydrophilicity of the film network, consequently diminishing its affinity for water vapor and adversely affecting the barrier efficacy [18]. These counteracting effects collectively contribute to the net increase in WVP observed with rising SE concentrations.

With progressive elevation of SE content in the film-forming system, the thickness of G-C–SE composite films demonstrated a concomitant increase (Table 3). In G3.1:C0.4 and G3.2:C0.3 film systems, the tensile strength (TS) exhibited a distinct pattern of initial enhancement and then a subsequent decline with the SE concentration increased, whereas the elongation at break (EAB) followed an inverse trend. The maximum value of TS was 13.97 ± 1.65 MPa with a 0.5% SE incorporation, because of the phenolic hydroxyl groups within SE enhances cross-linking within the film-forming matrix [31]. In contrast, the G3.3:C0.2 film-forming system showed a progressive reduction in TS with increasing SE addition, while the EAB displayed an upward trend, reaching its peak value (114.52 ± 6.09%) at 0.5% SE content. The pronounced TS reduction beyond this threshold likely stems from excessive cross-linking induced by high SE loading, which over-constrains molecular mobility in the polymer network. Such hyper-cross-linking is presumed to render the film network overly rigid and brittle, thereby diminishing its load-bearing ability under tensile stress and ultimately lowering mechanical strength [31].

#### 3.2.3. FTIR

**Figure 2:**
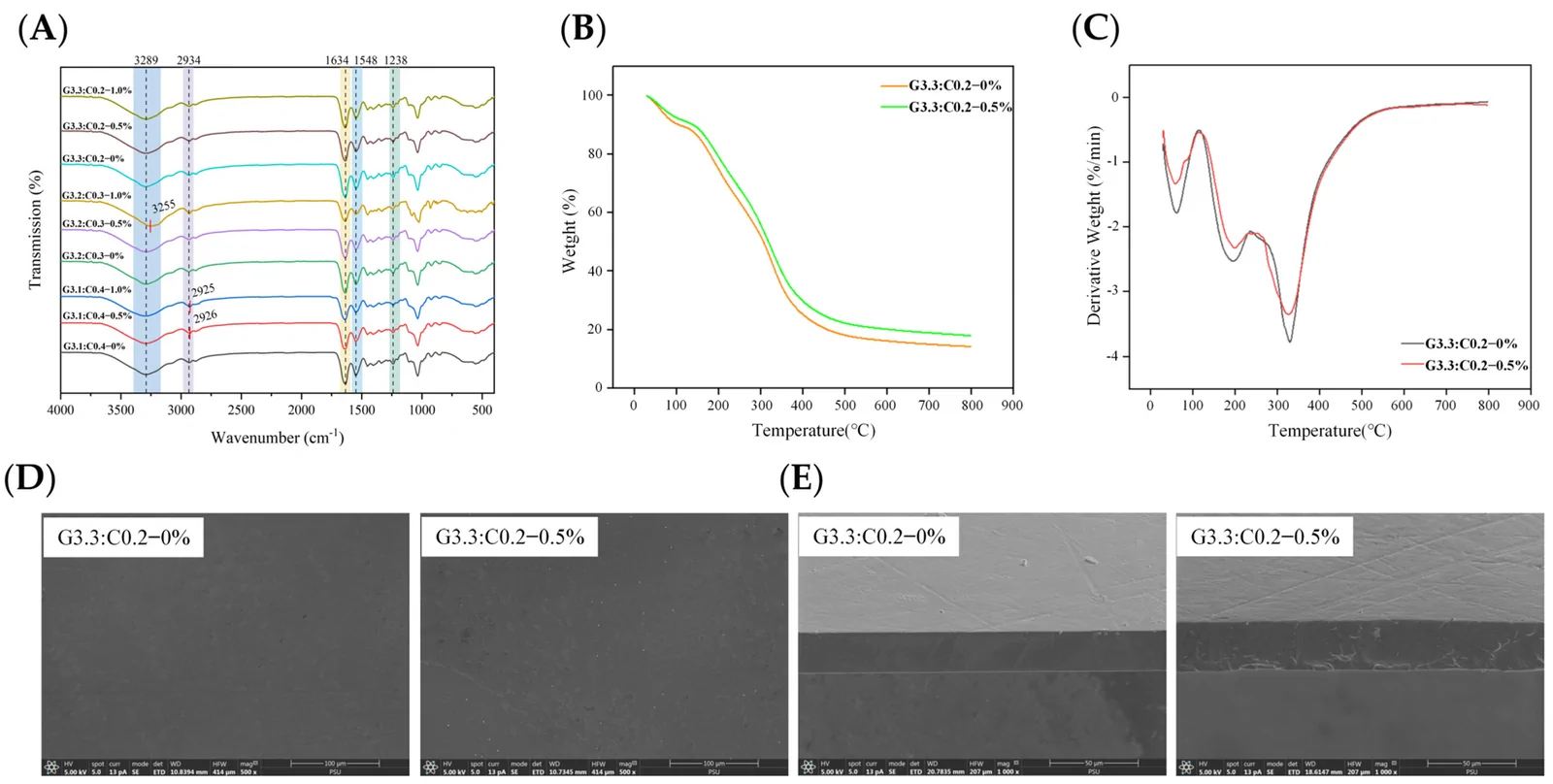
FTIR spectra (**A**), thermogravimetric analysis (TGA) plots (**B**), derivative thermogravimetry (DTG) plots (**C**), surface SEM (**D**) with 500 times magnification, cross-sectional SEM (**E**) with 1000 times magnification of the G-C–SE films. The vertical colored dotted lines indicate the characteristic peaks.

FTIR spectra revealed the structural features and intermolecular interaction modes within the film matrix. As depicted in Figure 2A, all film formulations exhibited a broad and intense absorption band in the region of 3000–3700 cm^−1^, which corresponds to the amide A band (3289 cm^−1^) arising from N–H and O–H stretching vibrations. This spectral feature is predominantly attributed to the abundant –NH_2_ and –OH groups present in gelatin and chitosan molecules, which are prone to form intermolecular hydrogen bonds. These groups are prone to form intermolecular hydrogen bonds, resulting in perturbations in the vibrational frequencies of these functional groups, giving rise to the characteristic absorption in this spectral region [32]. Upon incorporation of SE, a red shift of this absorption band toward lower wavenumbers was observed, particularly for the G3.2:C0.3−1.0% sample (3255 cm^−1^), because of the supply of the additional hydroxyl groups in *H. fusiformis* components. The low dosages of phenolic hydroxyl moieties tend to promote cross-linking within the film-forming matrix, which in turn enhances the tensile strength of the resulting films [16]. The absorption band within the 2900–3000 cm^−1^ range, designated as the amide B band (2934 cm^−1^), originates from asymmetric and symmetric stretching vibrations of C–H bonds. The progressive intensification of this peak with increasing SE content suggests enhanced intermolecular interactions within the composite system. Other characteristic peaks were also identified: the band at 1634 cm^−1^ corresponds to the amide I band, associated with C=O stretching vibrations; the peak at 1548 cm^−1^ is assignable to the amide II band, arising from N–H bending coupled with C–N stretching; and the band at 1238 cm^−1^ is attributed to the amide III band, derived from in-plane bending of C–N and N–H groups linked to the amide moiety. These absorption features are consistent with the typical amide bond signatures of gelatin–chitosan films. In summary, the primary mode of interaction between SE and the G-C film matrix is noncovalent, predominantly mediated through hydrogen bonding. Such interactions enhance the compatibility of the blended system, thereby contributing to improved tensile strength and maintaining favorable WVP performance.

In summary, the G3.3:C0.2−0.5% film formulation exhibited a considerably higher elongation at EAB (114.52 ± 6.09%) and a lower WVP coefficient (6.09 ± 0.13 × 10^−11^ g m^−1^ s^−1^ Pa^−1^), indicating superior flexibility and enhanced water vapor barrier performance. These attributes are particularly critical for the preservation of RTE-SC, given its moist and irregular surface morphology, which renders it susceptible to mechanical deformation during handling and storage. Furthermore, the G3.3:C0.2 matrix composition demonstrated favorable optical transparency while retaining adequate TS (8.04 ± 0.12 MPa) upon SE incorporation. Taking into account the trade-offs among TS, EAB, WVP, light transmittance, and color characteristics, the G3.3:C0.2−0.5% film was ultimately selected as the optimal formulation for subsequent preservation experiments on RTE-SC.

#### 3.2.4. TGA for Optimal Film

TGA and derivative thermogravimetric (DTG) profiling were conducted to evaluate the thermal stability of the fabricated film materials. As illustrated in Figure 2B, two film samples both exhibited analogous degradation patterns, characterized by two distinct weight-loss stages. The first stage, occurring within the temperature range of 0–100 °C, corresponds to the evaporation of moisture absorbed within the film matrix. During this phase, the G.3.1:C0.4−0% and G.3.1:C0.4−0.5% samples experienced a reduction of approximately 12%. The second degradation stage, spanning from 150 to 350 °C, accounted for a more substantial weight loss of about 65%, which was driven by the thermal decomposition of gelatin, partial breakdown of chitosan, and pyrolytic degradation of the incorporated SE constituents. Notably, the residual mass following thermal decomposition was approximately 14% for the SE-free formulation (G.3.1:C0.4−0%), whereas the G.3.1:C0.4−0.5% retained about 18% of its original mass. The inclusion of SE contributed to an increased char residue, implying enhanced thermal stability of the composite films.

The DTG curves (Figure 2C) revealed two prominent thermal transition zones for the G-C–SE films, occurring at approximately 200 °C and 330 °C, respectively. The former transition is presumably associated with the cleavage of intermolecular side-chain linkages, while the latter corresponds to the disruption of the main-chain backbone structure of the composite matrix. Two distinct maximum weight-loss rate peaks were discernible in the DTG profiles, corresponding to two thermal transition temperatures, designated as T_1_ and T_2_. Prior to T_1_, the degradation rate progressively increased with rising temperature, indicating the onset of structural breakdown. As the temperature approached T_2_, a second rupture event occurred, at which point the main-chain architecture was completely disrupted, manifesting as another peak in the weight-loss rate. The incorporation of SE led to a marked elevation in both T_1_ and T_2_ values, suggesting an appreciable improvement in thermal resistance. It is plausible that the added SE reinforces intermolecular interactions within the chitosan-based matrix, thereby conferring greater structural integrity at elevated temperatures.

#### 3.2.5. SEM for Optimal Film

Representative surface and cross-sectional micrographs of the various film formulations, are presented in Figure 2D,E. For the SE-free film formulation, the cross-section images revealed a smooth and homogeneous morphology, devoid of any detectable cracks, voids, or other structural irregularities, thereby indicating favorable compatibility and uniform dispersion within the matrix. Upon incorporation with SE, a progressive roughening of the cross-sectional topography was observed, accompanied by the emergence of slight protrusions on the film surface. The granular microstructures became discernible, because the encapsulating effect of chitosan induced by the presence of SE-derived components, potentially leading to localized aggregation. Despite these morphological alterations, all the composite films maintained uniform thickness and regular textural features, with no macroscopic phase separation.

### 3.3. The Application of the Optimal Film in Ambient Temperature Storage of RTE-SC

#### 3.3.1. Visual Appearance Changes of RTE-SC

**Figure 3:**
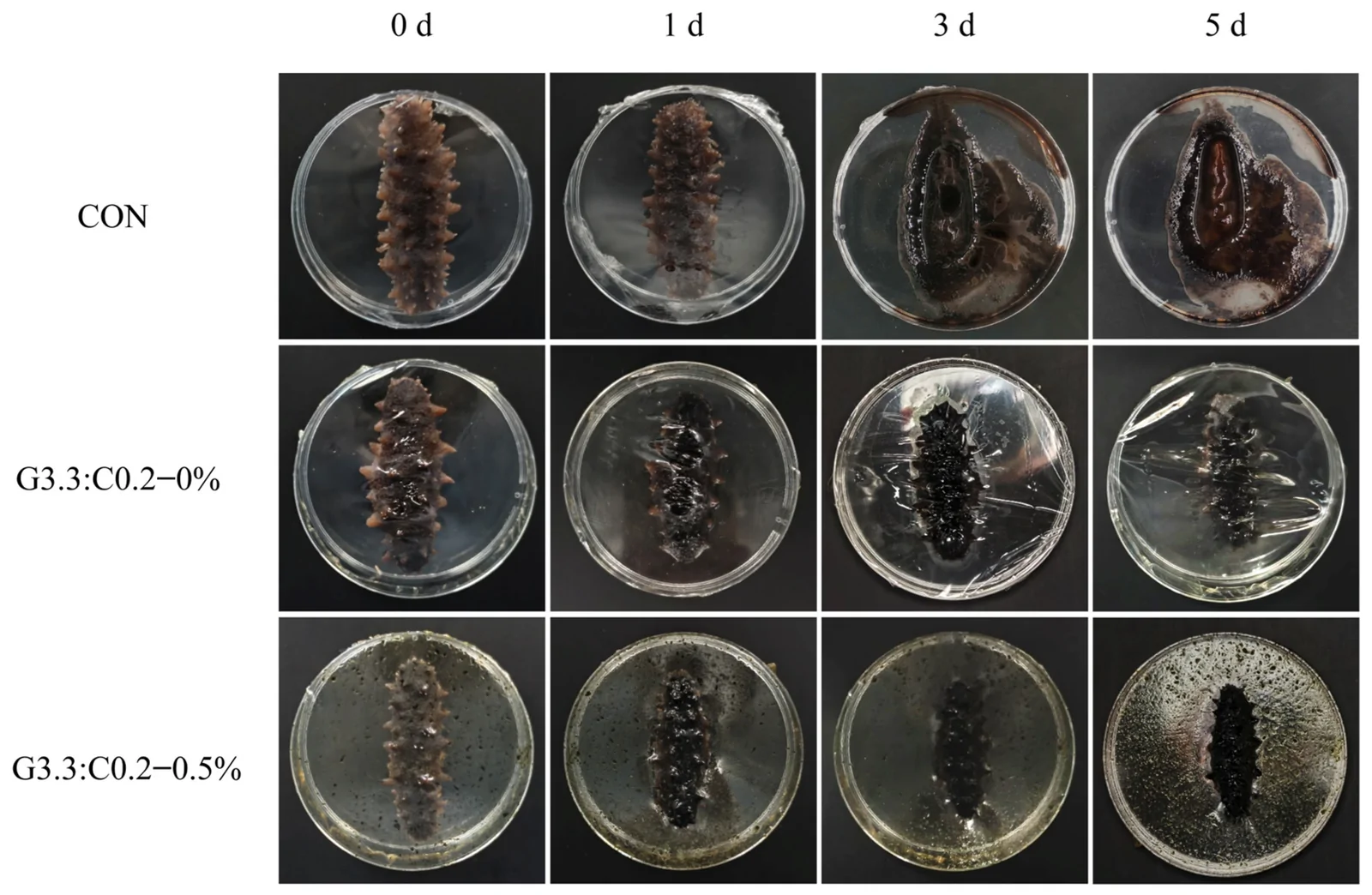
The visual appearance changes of RTE–SC during the 5 days of storage at room temperature.

The morphological alterations of RTE-SC samples during ambient conditions storage are illustrated in Figure 3. At the initial stage of storage, all three groups exhibited satisfactory visual quality, characterized by intact external morphology, plump body contours, erect papillae, firm and elastic textures, as well as favorable palatability. The specimens displayed a dark grayish coloration with no detectable off-odors, indicating good overall initial quality. On the third day of storage, the papillae on the RTE-SC in the CON group had gradually diminished, accompanied by the onset of surface deterioration. Especially, the RTE-SC of CON group had completely lost their original morphological integrity. This deterioration was presumably attributable to microbial proliferation on the surface of the RTE-SC, which triggered spoilage and putrefaction, concomitant with non-enzymatic degradation of collagen within the SCBW, resulting in collagen fiber fragmentation and an increase in soluble protein content [33].

Throughout the entire storage period, the G3.3:C0.2−0% group samples exhibited merely mild surface stickiness and slight browning, along with a visible shrinkage in overall appearance compared with the fresh sample. Conversely, the G3.3:C0.2−0.5% group displayed virtually no adhesion or discoloration, while maintaining firm elasticity without discernible visual differences from the CON group. The application of bioactive film wrapping effectively retarded the morphological degradation of sea cucumber samples, although a pronounced shrinkage was observed, the overall structural integrity largely preserved. The incorporation of SE (rich in carboxylic acid) plays a pivotal role in further suppressing microbial growth and reproduction, thereby delaying the spoilage and quality deterioration processes of the sea cucumber during storage [5].

#### 3.3.2. Textural Characteristics

TPA is regarded as a reliable indicator for characterizing structural alterations in food tissues, effectively reflecting the organizational integrity of the SCBW. As summarized in Table 4, prolonged storage duration led to a significant decline in hardness, cohesiveness, springiness, chewiness, and resilience across all three experimental groups (*p* < 0.05). At day 0, no statistically significant differences were observed among the three groups for any texture attributes (*p* > 0.05). After three days storage, the springiness results of the CON, G3.3:C0.2−0%, and G3.3:C0.2−0.5% groups exhibited declines of 62.78%, 50.31%, and 37.72%, respectively. Furthermore, the CON group displayed the greatest textural deterioration compared with fresh samples, with a decrease of 62.18%, 50.66%, 78.16%, and 64.72% to hardness, cohesiveness, chewiness, and resilience indicators, respectively. This substantial loss is primarily driven by the progressive degradation of collagen fibers within the SCBW.

Nevertheless, both the G3.3:C0.2−0% and G3.3:C0.2−0.5% groups maintained relatively favorable texture profiles after 5 days storage, suggesting that bioactive film coatings effectively modulated the rate of quality deterioration. This observation implies that the internal structural compactness of the SCBW was better preserved in the treated groups. Unfortunately, the CON group exhibited severe softening and extensive structural disintegration, the TPA measurements was not feasible. The G3.3:C0.2−0.5% group demonstrated significantly less loss in hardness, cohesiveness, springiness, chewiness, and resilience, with values superior to those of the G3.3:C0.2−0% group by margins of 34.16%, 44.71%, 22.82%, 23.40%, and 22.26%, respectively (*p* < 0.05). This protective effect is plausibly attributed to the phenolic compounds in the SE, which scavenge the free radicals and mitigate the protein degradation [10]. Collectively, the SE-enriched film formulation (G3.3:C0.2−0.5%) exerts a beneficial effect in delaying sea cucumber softening, thereby contributing to the preservation of springiness and palatable chewiness during ambient storage.

#### 3.3.3. TCA-Soluble Oligopeptides, and GAGs

**Figure 4:**
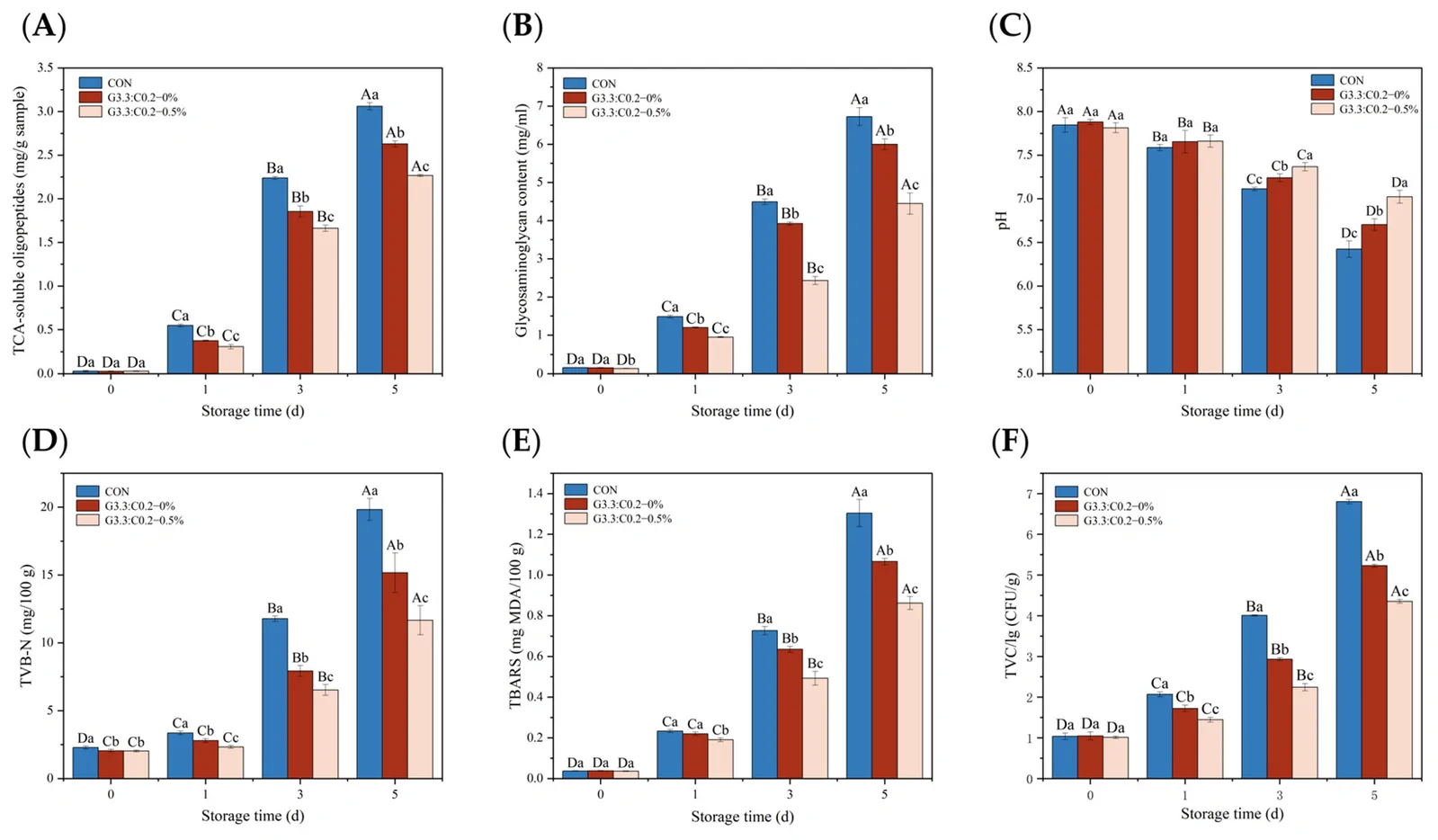
Changes in TCA-soluble oligopeptides (**A**), glycosaminoglycans content (GAGs) (**B**), pH (**C**), total volatile basic nitrogen (TVB-N) (**D**), thiobarbituric acid reactive substances (TBARS) (**E**), total viable count (TVC) (**F**) of RTE-SC stored at ambient temperature. Note: Superscript lowercase letters in the same column indicate significant intra-group differences (*p* < 0.05), and different superscript uppercase letters in the same column indicate significant differences between groups (*p* < 0.05).

The content of TCA-soluble oligopeptides is associated with protein degradation in aquatic products during processing and storage. This indicator not only affects textural properties and freshness but also reflects the dynamic changes in the low-molecular-weight peptide fractions [34]. At the beginning of storage, the TCA-soluble oligopeptide contents of the three sea cucumber groups ranged from 0.02 to 0.04 mg/g, with no significant differences among them (*p* > 0.05). However, a progressive increase in TCA-soluble oligopeptide content was observed in all treatment groups (Figure 4A). After 5 days of storage, the TCA-soluble oligopeptide contents increased significantly (*p* < 0.05) by 3.03, 2.60, and 2.24 mg/g for the respective groups. On the one hand, the continuous collagen degradation within the SCBW leads to loosening of the tissue matrix and reduced elasticity, sensory attributes and freshness [35]. On the other hand, the exogenous microbial enzymes promotes proteolytic breakdown, facilitating the progressive conversion of macromolecular collagen into smaller peptide fragments and free amino acids, which collectively contribute to the increased results [36].

The CON group consistently exhibited significantly higher TCA-soluble oligopeptide results than other groups (*p* < 0.05). The inferior gas permeability of PVC packaging material relative to the bioactive films, which resulted in the accumulation of water vapor from sea cucumber moisture evaporation, thereby creating a high-humidity microenvironment. In contrast, the G3.3:C0.2−0.5% group effectively suppressed proteolysis, as reflected by the lower generation of TCA-soluble oligopeptides and the protection of fiber structural integrity and overall quality of RTE-SC. This can be attributed to the presence of vanillic acid 4-sulfate in the *H. fusiforme* extract, which contains phenolic hydroxyl moieties capable of directly scavenging reactive oxygen species generated during storage. This action alleviates oxidative modifications of protein side chains induced by free radicals, mitigates the oxidative disruption of higher-order collagen fibril structures, and reduces peptide bond cleavage mediated by oxidative stress [10]. Moreover, brown algal polyphenols can interact with sea cucumber collagen through hydrogen bonding and hydrophobic interactions, thereby inducing moderate cross-linking of collagen molecules. Such cross-linking enhances the structural stability of collagen fibrils, while simultaneously shielding the cleavage sites of collagenases, reducing the substrate susceptibility of collagen to proteolytic enzymes, and consequently delaying the conversion of macromolecular collagen into oligopeptide fragments [37].

GAGs play a critical role in maintaining the structural integrity of collagen fibrils, which form proteoglycan complexes through covalent association with proteins. Therefore, the quantification of GAGs content serves as an effective parameter to detect the stability of the collagen architecture and the deterioration of SCBW [38]. With progressive storage period, the gathering of GAGs released from all three groups exhibited a consistent update tendency (Figure 4B). By the end of storage, the GAG contents had reached 6.72 ± 0.24 mg/mL, 6.00 ± 0.14 mg/mL, and 4.45 ± 0.28 mg/mL for the respective groups (CON, G3.3:C0.2−0%, G3.3:C0.2−0.5%), corresponding to 43.05-fold, 39.20-fold, and 32.66-fold to their initial values (*p* < 0.05). This substantial elevation is plausibly attributable to the cleavage of peptide bonds within the collagenous tissue matrix of the SCBW, leading to progressive liberation of GAGs in various forms, including polysaccharide fragments and low molecular weight soluble saccharides [35]. The GAGs released amount in the CON group were significantly higher than those recorded for the bioactive film protective groups (*p* < 0.05). This phenomenon is probable due to the absence of protection in the CON group, rendering the RTE-SC susceptible to protein oxidative degradation and peptide bond cleavage during the storage, which in turn facilitated the dissolution and loss of GAGs from the SCBW. Furthermore, the G3.3:C0.2−0.5% group exhibited the lowest GAGs content among all coatings, which might be ascribed to the potential antioxidation present in the SE consequently impeding the degradation and releasing of collagen fibrillar proteins during ambient storage. The LC-MS identification results (Table S2) further confirmed that the *H. fusiforme* extract contains a diverse array of bioactive substances, including phenolic derivatives and organic acids. The synergistic effects of these components endow the brown seaweed extract with favorable antioxidant properties, effectively inhibiting collagen structural deterioration in ready-to-eat sea cucumber induced by protein oxidation [39]. Among these, vanillic acid 4-sulfate—as a phenolic derivative—exhibits excellent free radical scavenging capacity. The radical-quenching activity of its parent compound, vanillic acid, can effectively neutralize reactive oxygen species accumulated during storage, thereby protecting amino acid residues within collagen fibrils from oxidative modification and significantly retarding oxidative peptide bond cleavage [40]. Meanwhile, organic acids such as citric acid and succinic acid function as natural metal ion chelators. By complexing transition metal ions present in the system, they interrupt oxidative radical chain reactions, further reinforcing the overall antioxidant efficacy [41]. This combined action alleviates oxidative damage to the collagen network within the sea cucumber body wall and reduces the leaching of GAGs.

#### 3.3.4. pH and TVB-N

The impact of various coatings on the pH changes of RTE-SC samples throughout the ambient storage is presented in Figure 4C. With the extended storage period, a clear declining in pH results was emerged within all experimental groups (*p* < 0.05). At the initial stage, the pH values of CON, G3.3:C0.2−0%, and G3.3:C0.2−0.5% groups were recorded as 7.85 ± 0.08, 7.88 ± 0.03, and 7.81 ± 0.06, respectively, with no statistically significant differences (*p* > 0.05). On the 3rd day, the pH of the CON group had descended dramatically below those of the other two groups (*p* < 0.05). At the end of the storage, the CON group exhibited the most pronounced decrease, with an 18% reduction relative to the initial value. This decrease of pH value is about to the limited antimicrobial activity and high gas permeability of PVC cling film, which creates a microenvironment conducive to microbial proliferation. The consequent enhancement of anaerobic glycolysis leads to the accumulation of lactic acid and other organic acids, which lowers the pH value. The decline in pH value can be attributed to the limited antimicrobial activity of the PVC cling film, combined with the high-humidity environment within the packaging, which collectively created a favorable microenvironment for microbial proliferation. During frozen storage, the protein- and polysaccharide-rich nutritional matrix of the SCBW itself provided abundant carbon and nitrogen sources for spoilage microorganisms. Following substantial microbial growth, acidic metabolic byproducts—including lactic acid and short-chain fatty acids—were generated through anaerobic metabolism, thereby contributing to the observed reduction in pH [42]. Moreover, the G3.3:C0.2−0.5% group exhibited the most stable pH profile, exhibiting only a 10% decline compared with the original result. This improved pH retention is likely associated with the antioxidant and antimicrobial properties of SE incorporated into the film matrix [43]. By mitigating protein oxidation and suppressing microbial spoilage, the SE-enriched film effectively retarded the degradation of sea cucumber collagen fibers and enhanced storage stability of the RTE-SC samples [5].

At the initial stage, the TVB-N values of the CON, G3.3:C0.2−0%, and G3.3:C0.2−0.5% groups were determined to be 2.29 ± 0.11 mg/100 g, 2.05 ± 0.11 mg/100 g, and 2.03 ± 0.07 mg/100 g, respectively. A progressive increase in TVB-N content was observed across all groups (*p* < 0.05) with prolonged storage duration, thus all measurements remained below the regulatory threshold of 20 mg/100 g. The CON group exhibited the most rapid escalation in TVB-N results, reaching a maximum of 19.83 ± 0.81 mg/100 g after the 5 days storage. This phenomenon can be ascribed to the secretion of substantial amounts of extracellular proteases by spoilage microorganisms during storage, which accelerated the hydrolysis of collagen and proteoglycans within the SCBW. This enzymatic degradation promoted the extensive generation of TCA-soluble oligopeptides and TVB-N, thereby accelerating the structural disintegration and quality deterioration of the collagen tissue. In comparison, the G3.3:C0.2−0% group and G3.3:C0.2−0.5% group demonstrated a slower TVB-N content, which was significantly lower than that of CON group at day 5 (*p* < 0.05), demonstrating that the bioactive film packaging conferred superior preservative efficacy relative to conventional PVC film. Firstly, the low light transmittance of the SE-enriched film effectively attenuated ultraviolet radiation (Figure 1), thereby minimizing photo-induced oxidative damage to the SCBW. The bioactive constituents present in SE exert antimicrobial effects by compromising bacterial cell membrane integrity and interfering with microbial metabolic pathways. The SE-loaded preservative film forms a physical barrier layer on the surface of sea cucumber specimens, which not only reduces microbial adhesion and invasion at the physical level but also enables the sustained release of active components—such as brown algal polyphenols—onto the sample surface. This dual action effectively inhibits the growth and proliferation of spoilage microorganisms, thereby reducing the total microbial load [44]. Concurrently, it interferes with the metabolic pathways of spoilage bacteria, downregulates the synthesis and secretion of extracellular proteases, attenuates the microbe-mediated proteolytic degradation processes, and mitigates the accumulation of TVB-N during storage [45].

#### 3.3.5. TBARS and TVC

As shown in Figure 4E, a sustaining elevation in TBARS results was captured across all experimental groups via extension of storage period, which accounted for the unsaturated fatty acids present in the SCBW underwent continuous chain oxidation under the combined effects of oxygen, light exposure, metal ions, and residual endogenous enzymes, thereby promoting lipid peroxidation and degradation within the sea cucumber matrix [5]. The malondialdehyde (MDA) contents of the three groups ranged from 0.35 to 0.39 mg MDA/100 g without significant differences (*p* > 0.05). On the 5th day, the MDA contents of the CON, G3.3:C0.2−0%, and G3.3:C0.2−0.5% groups had increased by 34.96-fold, 28.01-fold, and 23.68-fold, respectively, relative to their initial numeric values. The superior performance of the G3.3:C0.2−0.5% film can be reasonably attributed to the phenolic hydroxyl moieties present in the incorporated SE, which are capable of donating electrons to quench free radicals during storage, thereby interrupting the oxidative chain reaction of lipids [46]. This mechanism effectively retarded the progression of lipid oxidation and contributed to the suppression of quality deterioration in the RTE-SC products.

The evolution of TVC in RTE-SC samples was emerged in Figure 4F. The initial TVC values of the CON, G3.3:C0.2−0%, and G3.3:C0.2−0.5% groups were 1.04 ± 0.08, 1.05 ± 0.10, and 1.01 ± 0.03 lg (CFU/g), respectively (*p* > 0.05). Nevertheless, the accompanying with the storage duration, the CON group exhibited the fastest increase in TVC, reaching a peak value of 6.80 ± 0.05 lg (CFU/g) on the 5th day storage. The G3.3:C0.2−0% group recorded a TVC of 5.23 ± 0.04 lg (CFU/g) at the same time, both of which exceeded the national regulatory limitation of 5.00 lg (CFU/g). At this stage, the SCBW had undergone substantial spoilage, characterized by loss of original morphology, softening of the body wall, and development of off-odors (see Figure 1). In contrast, the TVC value of G3.3:C0.2−0.5% group was merely 4.35 ± 0.05 lg(CFU/g), which remained within the acceptable range. These findings emphasizes that the SE-loaded active film exerted considerably superior preservative effects, effectively extending the shelf-life of RTE-SC reposited at ambient temperature by approximately 2–3 days. This enhanced antimicrobial efficacy is plausibly attributable to multiple factors. Firstly, chitosan, as a primary constituent of the film-forming matrix, possesses inherent antibacterial activity, which facilitates penetration of bacterial cell walls and effectively suppresses microbial proliferation, demonstrating markedly better performance than conventional PVC cling film. Secondly, the presence of polyphenolic compounds in the SE is considered to contribute synergistically to the antimicrobial effect. These phenolic constituents might exert their inhibitory action through multiple mechanisms, including disruption of bacterial membrane permeability, inactivation of key intracellular enzymes, and specific binding interactions with membrane-associated components or adhesion-related molecules on the bacterial surface [29].

## 4. Conclusions

In this study, a gelatin/chitosan active composite film incorporated with SE was successfully fabricated and demonstrated to exert a remarkable preservative effect on RTE-SC ambient storage. SE is rich in bioactive constituents such as organic acids and phenolic compounds, imparted favorable antioxidant and antimicrobial properties to the film matrix, which can effectively enhance the mechanical strength, WVP performance, and thermal stability of the composite films, yielding a structurally homogeneous active packaging material. When applied to the preservation of RTE-SC, the G-C–SE film substantially retarded the quality deterioration process via its multifunctional active properties. Specifically, it effectively suppressed protein degradation and lipid oxidation, as evidenced by the controlled elevation of TVB-N and TBARS values. The protection of film coatings effectively preserved the desirable textural attributes and visual appearance throughout the storage period. The SE-loaded gelatin/chitosan composite film demonstrates a green and highly efficient active packaging solution. This coating offers a feasible technological approach for the ambient-temperature preservation of RTE-SC, exhibiting promising potential for extending shelf life while sustaining product quality. Nevertheless, certain limitations should be acknowledged in the present study. The experiments were conducted exclusively under constant ambient temperature conditions, without simulating the complex storage and transportation environments encountered in actual distribution channels, such as temperature fluctuations and high humidity. In addition, the current investigation primarily focused on conventional physicochemical quality indicators, with relatively limited exploration of the structural alterations in sea cucumber proteins at the molecular level or the dynamic succession of microbial communities during storage. Future work should therefore be directed toward application validation under real-world logistics scenarios to better reflect practical conditions. Combined with multi-omics approaches, further studies are warranted to elucidate the molecular and microbial mechanisms underlying the preservative effects of the coating treatment. Moreover, optimization of the film-forming process, along with systematic evaluation of its safety and degradation characteristics, will be essential to provide more comprehensive data support for the industrial-scale application of this active coating technology.

## Figures and Tables

**Table 1:** TPC, TFC contents and antioxidant activities of *Hizikia fusiforme*/*Laminaria japonica* extracts prepared with different ethanol concentrations.

Sample	Ethanol Concentration (%)	Extraction Yield (%)	TPC /(mg/g)	TFC /(mg/g)	Analysis of Antioxidant			Antimicrobial Properties			
					DPPH /(μmol/g)	ABTS /(μmol/g)	FRAP /(μmol/g)	*S. aureus*		*E. coli*	
								MIC /(mg/mL)	MBC /(mg/mL)	MIC /(mg/mL)	MBC /(mg/mL)
*Hizikia fusiformis*	60	3.16 ± 0.15 ^Ca^	16.40 ± 0.24 ^Aa^	141.25 ± 4.51 ^Aa^	184.26 ± 0.33 ^Aa^	1133.01 ± 17.94 ^Aa^	10.81 ± 0.22 ^Aa^	1.56	6.25	50	50
	70	2.78 ± 0.19 ^Ea^	11.81 ± 0.29 ^Bb^	142.92 ± 2.60 ^Aa^	116.82 ± 1.30 ^Bb^	734.97 ± 16.81 ^Bb^	10.18 ± 0.22 ^Bb^	-	-	-	-
	80	1.67 ± 0.31 ^Db^	8.57 ± 0.15 ^Cc^	144.58 ± 1.91 ^Aa^	68.66 ± 7.67 ^Cc^	597.36 ± 20.50 ^Cc^	7.64 ± 0.25 ^Cc^	-	-	-	-
*Laminaria* *japonica*	60	6.26 ± 0.17 ^Aa^	6.06 ± 0.15 ^Da^	83.33 ± 4.02 ^Ba^	27.57 ± 2.14 ^Da^	579.60 ± 14.27 ^Ca^	7.83 ± 0.22 ^Ca^	6.25	25	25	50
	70	5.33 ± 0.22 ^Bb^	5.65 ± 0.13 ^Eb^	78.75 ± 3.75 ^Ba^	12.27 ± 2.99 ^Fc^	419.79 ± 9.24 ^Db^	5.00 ± 0.29 ^Db^	-	-	-	-
	80	2.92 ± 0.13 ^CDc^	4.92 ± 0.15 ^Fc^	79.58 ± 5.64 ^Ba^	20.49 ± 1.70 ^Eb^	370.96 ± 10.25 ^Ec^	3.35 ± 0.08 ^Ec^	-	-	-	-

Note: Values are mean ± standard deviation (*n* = 3). Superscript lowercase letters in the same column indicate significant intra-group differences (*p* < 0.05), and different superscript uppercase letters in the same column indicate significant differences between groups (*p* < 0.05).

**Table 2:** Color parameters of G-C–SE films.

Samples	L*	a*	b*	c*	H*	*Δ* *E*
G3.1:C0.4−0%	88.71 ± 0.20 ^a^	−2.23 ± 0.03 ^c^	1.65 ± 0.14 ^g^	1.53 ± 0.12 ^g^	−0.64 ± 0.04 ^d^	4.41 ± 0.17 ^g^
G3.1:C0.4−0.5%	73.91 ± 0.68 ^cd^	−4.81 ± 0.54 ^e^	46.45 ± 0.48 ^e^	46.12 ± 0.45 ^e^	−1.47 ± 0.01 ^f^	49.86 ± 0.65 ^e^
G3.1:C0.4−1.0%	65.68 ± 0.57 ^e^	0.54 ± 0.55 ^b^	58.92 ± 0.31 ^b^	58.47 ± 0.31 ^b^	1.56 ± 0.001 ^a^	64.48 ± 0.34 ^b^
G3.2:C0.3−0%	89.21 ± 0.26 ^a^	−1.95 ± 0.04 ^c^	0.90 ± 0.06 ^g^	0.82 ± 0.03 ^g^	−0.43 ± 0.03 ^b^	3.72 ± 0.25 ^g^
G3.2:C0.3−0.5%	74.06 ± 0.83 ^d^	−4.99 ± 0.13 ^d^	49.85 ± 0.93 ^c^	49.53 ± 0.93 ^d^	−1.47 ± 0.002 ^f^	52.97 ± 1.16 ^c^
G3.2:C0.3−1.0%	63.99 ± 0.76 ^b^	1.35 ± 0.14 ^f^	66.20 ± 1.08 ^f^	65.78 ± 1.09 ^a^	1.55 ± 0.002 ^a^	71.83 ± 0.87 ^f^
G3.3:C0.2−0%	88.71 ± 0.78 ^a^	−2.12 ± 0.03 ^c^	1.32 ± 0.04 ^g^	1.22 ± 0.23 ^g^	−0.56 ± 0.02 ^c^	4.31 ± 0.75 ^g^
G3.3:C0.2−0.5%	72.76 ± 0.83 ^c^	−4.28 ± 0.3 ^e^	52.07 ± 0.28 ^d^	51.69 ± 0.30 ^c^	−1.49 ± 0.005 ^f^	55.45 ± 0.11 ^d^
G3.3:C0.2−1.0%	78.92 ± 0.90 ^f^	−6.43 ± 0.08 ^a^	41.35 ± 1.28 ^a^	41.20 ± 1.27 ^f^	−1.42 ± 0.004 ^e^	43.50 ± 1.00 ^a^

Note: Values are mean ± standard deviation (*n* = 3). Superscript lowercase letters in the same column indicate significant intra-group differences (*p* < 0.05).

**Table 3:** Physical and mechanical properties of G-C–SE films.

Samples	Thickness/(mm)	TS/(MPa)	EAB/(%)	WVP /(×10^−11^ g m^−1^ s^−1^ Pa^−1^)
G3.1:C0.4−0%	0.026 ± 0.002 ^abc^	6.87 ± 0.61 ^bc^	83.67 ± 2.73 ^d^	5.91 ± 0.10 ^e^
G3.1:C0.4−0.5%	0.026 ± 0.002 ^bc^	13.97 ± 1.65 ^a^	82.3 ± 3.52 ^d^	6.77 ± 0.10 ^cd^
G3.1:C0.4−1.0%	0.027 ± 0.001 ^abc^	7.96 ± 0.17 ^b^	95.8 ± 3.83 ^c^	7.21 ± 0.32 ^b^
G3.2:C0.3−0%	0.023 ± 0.003 ^d^	5.92 ± 0.34 ^cd^	81.5 ± 3.66 ^d^	6.51 ± 0.15 ^d^
G3.2:C0.3−0.5%	0.026 ± 0.003 ^cd^	7.05 ± 0.73 ^bc^	80.5 ± 1.92 ^d^	6.84 ± 0.13 ^c^
G3.2:C0.3−1.0%	0.028 ± 0.002 ^ab^	5.32 ± 0.84 ^d^	105.1 ± 5.28 ^b^	7.65 ± 0.14 ^a^
G3.3:C0.2−0%	0.027 ± 0.004 ^abc^	8.36 ± 0.90 ^b^	63.13 ± 2.38 ^e^	5.95 ± 0.15 ^e^
G3.3:C0.2−0.5%	0.025 ± 0.002 ^cd^	8.04 ± 0.12 ^b^	114.52 ± 6.09 ^a^	6.09 ± 0.13 ^e^
G3.3:C0.2−1.0%	0.029 ± 0.002 ^a^	8.09 ± 0.28 ^b^	109.06 ± 0.56 ^ab^	7.75 ± 0.12 ^a^

Note: Values are mean ± standard deviation (*n* = 6). Superscript lowercase letters in the same column indicate significant intra-group differences (*p* < 0.05).

**Table 4:** TPA parameters including hardness, cohesiveness, springiness, chewiness and resilience values of sea cucumber in different storage times at room temperature.

Storage Period(Days)	Sample	Hardness/(N)	Cohesiveness	Springiness	Chewiness/(mJ)	Resilience
0	CON	12.25 ± 1.03 ^Aa^	0.82 ± 0.06 ^Aa^	1.55 ± 0.24 ^Aa^	15.38 ± 0.31 ^Ab^	0.77 ± 0.03 ^Aa^
	G3.3:C0.2−0%	12.63 ± 0.40 ^Aa^	0.83 ± 0.03 ^Aa^	1.75 ± 0.03 ^Aa^	16.18 ± 0.30 ^Aa^	0.79 ± 0.02 ^Aa^
	G3.3:C0.2−0.5%	12.3 ± 0.18 ^Aa^	0.81 ± 0.01 ^Aa^	1.64 ± 0.06 ^Aa^	15.75 ± 0.20 ^Aab^	0.77 ± 0.01 ^Aa^
1	CON	7.58 ± 0.39 ^Bc^	0.61 ± 0.03 ^Bc^	1.21 ± 0.11 ^Bb^	8.24 ± 0.28 ^Bb^	0.46 ± 0.07 ^Bb^
	G3.3:C0.2−0%	8.98 ± 0.80 ^Bb^	0.68 ± 0.02 ^Bb^	1.53 ± 0.12 ^Ba^	11.86 ± 1.24 ^Ba^	0.63 ± 0.04 ^Ba^
	G3.3:C0.2−0.5%	10.78 ± 0.74 ^Ba^	0.75 ± 0.03 ^Ba^	1.55 ± 0.08 ^Ba^	12.29 ± 0.42 ^Ba^	0.63 ± 0.01 ^Ba^
3	CON	4.63 ± 0.42 ^Cc^	0.40 ± 0.04 ^Cc^	0.58 ± 0.03 ^Cc^	3.36 ± 0.28 ^Cc^	0.27 ± 0.03 ^Cc^
	G3.3:C0.2−0%	6.52 ± 0.35 ^Cb^	0.58 ± 0.05 ^Cb^	0.87 ± 0.04 ^Cb^	6.11 ± 0.52 ^Cb^	0.57 ± 0.03 ^Cb^
	G3.3:C0.2−0.5%	8.87 ± 0.42 ^Ca^	0.66 ± 0.02 ^Ca^	1.02 ± 0.07 ^Ca^	7.24 ± 0.52 ^Ca^	0.61 ± 0.03 ^Ba^
5	CON	-	-	-	-	-
	G3.3:C0.2−0%	3.87 ± 0.36 ^Db^	0.34 ± 0.04 ^Db^	0.50 ± 0.05 ^Db^	3.54 ± 0.46 ^Db^	0.52 ± 0.04 ^Da^
	G3.3:C0.2−0.5%	6.53 ± 0.37 ^Da^	0.54 ± 0.03 ^Da^	0.67 ± 0.03 ^Da^	6.07 ± 0.27 ^Da^	0.56 ± 0.04 ^Ca^

Note: Values are mean ± standard deviation (*n* = 6). Superscript lowercase letters in the same column indicate significant intra-group differences (*p* < 0.05), and different superscript uppercase letters in the same column indicate significant differences between groups (*p* < 0.05).

## Data Availability

The original contributions presented in this study are included in the article/supplementary material. Further inquiries can be directed to the corresponding author.

## Supplementary Materials

The following supporting information can be downloaded at: https://www.mdpi.com/article/10.3390/foods15183246/s1, Figure S1: LC-MS chromatographic profiles of *H. fusiforme* extract (a) and potential functional compounds (b–i); Figure S2: LC-MS chromatographic profiles of *L. japonica* extract (a) and potential functional compounds (b–f); Table S1: Compositions of gelatin chitosan active composite films with different seaweed extract (SE) incorporation levels; Table S2: LC-MS identification of components in *H. fusiforme* extract; Table S3: LC-MS identification of components in *L. japonica* extract.

## References

[1] Pangestuti R., Arifin Z. (2018). Medicinal and health benefit effects of functional sea cucumbers. J. Tradit. Complement. Med..

[2] Bureau of Fisheries and Fishery Administration, Ministry of Agriculture and Rural Affairs of the People’s Republic of China, National Fisheries Technology Extension Center, China Society of Fisheries (2025). China Fishery Statistics Yearbook.

[3] Zhu L.L., Qi X., Bai J., Sun X., Hou H. (2022). The mechanism of molecular cross-linking against nonenzymatic degradation in the body wall of ready-to-eat sea cucumber. Food Chem..

[4] Sun J.H., Li Y.M., Yan T.T., Yang J.F. (2024). Preparation of antibacterial composite film based on arginine-modified chitosan and its application in the preservation of ready-to-eat sea cucumber. Int. J. Biol. Macromol..

[5] Liu Z.Q., Li D.Y., Song L., Liu Y.X., Yu M.M., Zhang M., Rakariyatham K., Zhou D.Y., Shahidi F. (2020). Effects of proteolysis and oxidation on mechanical properties of sea cucumber (*Stichopus japonicus*) during thermal processing and storage and their control. Food Chem..

[6] Xiong X., Zhang C., Li H.Y., Qi H., Xu Z., Jiang Y., Dong X.F. (2025). Dual role of calcium in regulating collagen integrity and proteolytic response during sea cucumber *Apostichopus japonicus* tenderization. Food Res. Int..

[7] Hung Y.H.R., Chang Y.H., Lin H.J., Chiang L.H., Lin H.T.V. (2025). Enhanced antioxidant activity of brown seaweed *Laminaria japonica* by fermentation using isolated *Bacillus subtilis*. Bioresour. Bioprocess..

[8] Guo Y.C., Ming Y., Li X., Sun C.H., Dong X.P., Qi H. (2024). Effect of phlorotannin extracts from *Ascophyllum nodosum* on the textural properties and structural changes of *Apostichopus japonicus*. Food Chem..

[9] Jang H., Lee J., Park Y.K., Lee J.Y. (2024). Exploring the health benefits and concerns of brown seaweed consumption: A comprehensive review of bioactive compounds in brown seaweed and its potential therapeutic effects. J. Agric. Food Res..

[10] Ming Y., Wang Y.Z., Xie Y.Q.Q., Dong X.P., Nakamura Y., Chen X., Qi H. (2023). Polyphenol extracts from *Ascophyllum nodosum* protected sea cucumber (*Apostichopus japonicas*) body wall against thermal degradation during tenderization. Food Res. Int..

[11] Tagrida M., Benjakul S. (2021). Betel (*Piper betle* L.) leaf ethanolic extracts dechlorophyllized using different methods: Antioxidant and antibacterial activities, and application for shelf-life extension of Nile tilapia (*Oreochromis niloticus*) fillets. RSC Adv..

[12] Amin M., Temdee W., Buatong J., Odeyemi A.O., Benjakul S. (2025). Antimicrobial activities of ethanolic Kiam (*Cotyleobium lanceotatum*) wood extract and its application to extend shelf life of refrigerated Pacific white shrimp. J. Food Sci. Tech..

[13] Ahmad A.S., Sae-leaw T., Zhang B., Benjakul S. (2024). Antioxidant and antimicrobial activities of Ethanolic Jik (*Barringtonia acutangula*) leaf extract and its application for shelf-life extension of Pacific white shrimp meat during refrigerated storage. Food Control.

[14] Chen H.Q., Zhong Q.X. (2022). Physical and antimicrobial properties of self-emulsified nanoemulsions containing three synergistic essential oils. Int. J. Food Microbiol..

[15] Mutlu N. (2023). Effects of grape seed oil nanoemulsion on physicochemical and antibacterial properties of gelatin-sodium alginate film blends. Int. J. Biol. Macromol..

[16] Likhar V., Ponnusamy A., Singh A., Zhang B., Yin T., Kim J.T., Benjakul S. (2026). L-cysteine modified carbon dots from Duea ching (*Ficus botryocarpa* Miq.) fruit: A multifunctional nanomaterial in active chitosan/gelatin films for enhancing the shelf life of *Asian seabass* slices. Food Hydrocoll..

[17] Ranjbaryan S., Pourfathi B., Almasi H. (2019). Reinforcing and release controlling effect of cellulose nanofiber in sodium caseinate films activated by nanoemulsified cinnamon essential oil. Food Packag. Shelf Life.

[18] Arora D., Saini C.S. (2026). Bio-nanocomposite film based on pectin from citrus sinensis peel reinforced with montmorillonite with improved mechanical and barrier properties. Future Postharvest Food.

[19] Awal M.S., Benjakul S., Prodpran T., Nilsuwan K. (2025). Properties of emulsion co-precipitated collagen/bambara groundnut protein-based film as influenced by basil essential oil and soy lecithin. Polymers.

[20] Li T., Guo L., Yang K., Guo Y., Wang X.H., Li J. (2026). High UV-shielding multifunctional nanocomposite films based on polyvinyl alcohol/gelatin incorporated with carbon dots and citral-Pickering emulsion for cherry tomato preservation. Innov. Food Sci. Emerg. Technol..

[21] Shan P., Wang K., Yu F.Y., Yi L.Z., Sun L.P., Li H. (2023). Gelatin/sodium alginate multilayer composite film crosslinked with green tea extract for active food packaging application. Colloids Surf. A.

[22] Du Y.N., Li A.T., Yan J.N., Jiang X.Y., Wu H.T. (2021). Inhibitory effect of coelomic fluid isolates on autolysis of minced muscle tissue from sea cucumber *Stichopus japonicus*. J. Food Meas. Charact..

[23] Liu Z.Q., Liu Y.X., Zhou D.Y., Liu X.Y., Dong X.P., Li D.M., Shahidi F. (2019). The role of matrix metalloprotease (MMP) to the autolysis of sea cucumber (*Stichopus japonicus*). J. Sci. Food Agric..

[24] (2016). Determination of Volatile Basic Nitrogen in Food.

[25] (2016). Determination of Malondialdehyde in Food.

[26] (2022). Food Microbiology Examination, Determination of Colony Count.

[27] Aadil K.R., Barapatre A., Sahu S., Jha H., Tiwary B.N. (2014). Free radical scavenging activity and reducing power of *Acacia nilotica* wood lignin. Int. J. Biol. Macromol..

[28] Raudonis R., Raudone L., Jakstas V., Janulis V. (2012). Comparative evaluation of post-column free radical scavenging and ferric reducing antioxidant power assays for screening of antioxidants in strawberries. J. Chromatogr. A.

[29] Shahrier J., Rasul G., Afrin F., Islam R., Shah A.K.M.A. (2023). Extension of shelf life of Nile tilapia (*Oreochromis niloticus*) fillets using seaweed extracts during refrigerated storage. Food Sci. Nutr..

[30] Gómez-Estaca J., Gómez-Guillén M.C., Fernández-Martín F., Montero P. (2011). Effects of gelatin origin, bovine-hide and tuna-skin, on the properties of compound gelatin–chitosan films. Food Hydrocoll..

[31] Yuan B., Cao Y.X., Tang Q., Yuan Z.Q., Zhou Y.R., McClements D.J., Cao C.J. (2019). Enhanced performance and functionality of active edible films by incorporating tea polyphenols into thin calcium alginate hydrogels. Food Hydrocoll..

[32] May C., Gulsah K., Oscar Z., Tosif M.M., Goksen G. (2026). Multi-component chitosan–pectin films reinforced with *Padina pavonica* nanocellulose and *Thymus algeriensis* essential oil for red meat shelf-life enhancement. Food Chem. X.

[33] Wang J., Lin L., Sun X., Hou H. (2020). Mechanism of sea cucumbers (*Apostichopus japonicus*) body wall changes under different thermal treatment at micro-scale. LWT-Food Sci. Technol..

[34] Wang H.X., Ding F.Y., Ma L., Zhang Y.H. (2021). Edible films from chitosan-gelatin: Physical properties and food packaging application. Food Biosci..

[35] Sun X., Zhu L.L., Qi X., Zhang H.W., Wu L., Wang J.H., Hou H. (2022). Cleavage sites and non-enzymatic self-degradation mechanism of ready-to-eat sea cucumber during storage. Food Chem..

[36] Dong X.F., He B.Y., Jiang D., Yu C.X., Zhu B.w., Qi H. (2019). Proteome analysis reveals the important roles of protease during tenderization of sea cucumber *Apostichopus japonicus* using iTRAQ. Food Res. Int..

[37] Han Y.Q., Jiang J., Li J.J., Zhao L., Xi Z.H. (2024). Influences of polyphenols on the properties of crosslinked acellular fish swim bladders: Experiments and molecular dynamic simulations. Polymers.

[38] Liu Z.Q., Zhou D.Y., Liu Y.X., Yu M.M., Shahidi F. (2020). Inhibitory effect of natural metal ion chelators on the autolysis of sea cucumber (*Stichopus japonicus*) and its mechanism. Food Res. Int..

[39] Li Y.J., Fu X.T., Duan D.L., Liu X.Y., Xu J.C., Gao X. (2017). Extraction and Identification of Phlorotannins from the Brown Alga, *Sargassum fusiforme* (Harvey) Setchell. Mar. Drugs.

[40] Lahmidi S., Anouar E.H., El Hafi M., El-Guourrami O., Essassi E.M., Benzeid H., Alharthi A.I., Mague J.T. (2021). Synthesis, structural elucidation, and antioxidant activity of new phenolic derivatives containing piperidine moiety: Experimental and theoretical investigations. J. Heterocycl. Chem..

[41] Medeiros M.L., Cordeiro A.M.M.T., Queiroz N., Soledade L.E.B., Souza A.L., Souza A.G. (2013). Efficient antioxidant formulations for use in biodiesel. Energy Fuels.

[42] Yang J.J., Huang P.Y., Sun B.L., Yang W.G., Ou C.R., Yuan C.H., Huang T., Wei H.M. (2024). Comparison of freezing and heating treatment sequence on biochemical properties and flavor of swimming crabs (*Portunus trituberculatus*) meat during freeze-thaw cycles. Food Res. Int..

[43] Udomwasinakun N., Pirak T., Chanput W.P. (2022). Identification of polyphenols in white mugwort (*Artemisia lactiflora* Wall.) ethanolic extracts and their anti-inflammatory and anti-adipogenic activity potential. Food Biosci..

[44] Kadam S.U., Pankaj S.K., Tiwari B.K., Cullen P.J., O’Donnell C.P. (2015). Development of biopolymer-based gelatin and casein films incorporating brown seaweed *Ascophyllum nodosum* extract. Food Packag. Shelf Life.

[45] Wu J., Li C.S., Li L.G., Yang X.Q., Wang Y.Q., Zhou W.G. (2022). Improved physicochemical properties and product characteristics of tilapia surimi by tea polyphenols during chilled storage. LWT-Food Sci. Technol..

[46] Alirezaei Y., Soltanizadeh N., Fanaei M., Kaykha M.E.H. (2026). Green preservation: Enhancing shelf life of chicken meat using antimicrobial and antioxidant deep eutectic solvent nanoemulsions. Food Chem. X.

