# Supplementary material for: Application of Gelatin/Chitosan Films Loaded with Seaweed Extract of Ready-to-Eat Sea Cucumbers Preservation During Ambient-Temperature Storage
Source: Foods. 2026 Sep 14;15(18):3246. doi: 10.3390/foods15183246 (PMC13605880; doi:10.3390/foods15183246)
Supplement: Supplementary file 1 [file foods-15-03246-s001.zip › foods-4520906-supplementary.pdf]

Table S1 Compositions of gelatin-chitosan active composite films with different seaweed extract (SE) incorporation levels

Sample	Gelatin (G)	Chitosan (C)	SE
G3.1:C0.4-0%	3.1 wt%	0.4 wt%	0% (w/w)
G3.1:C0.4-0.5%	3.1 wt%	0.4 wt%	0.5% (w/w)
G3.1:C0.4-1.0%	3.1 wt%	0.4 wt%	1.0% (w/w)
G3.2:C0.3-0%	3.2 wt%	0.3 wt%	0% (w/w)
G3.2:C0.3-0.5%	3.2 wt%	0.3 wt%	0.5% (w/w)
G3.2:C0.3-1.0%	3.2 wt%	0.3 wt%	1.0% (w/w)
G3.3:C0.2-0%	3.3 wt%	0.2 wt%	0% (w/w)
G3.3:C0.2-0.5%	3.3 wt%	0.2 wt%	0.5% (w/w)
G3.3:C0.2-1.0%	3.3 wt%	0.2 wt%	1.0% (w/w)

Table S2 LC-MS identification of components in *H. fusiforme* extract

Peak no.	Identified compounds	Formula	m/z	Mass (g/mol)
1	D-Sorbitol	$C_6H_{14}O_6$	181.07	182.08
**2**	**Glyceric acid**	$C_3H_6O_4$	**105.02**	**106.03**
3	Uridine monophosphate (UMP)	$C_9H_{13}N_2O_9P$	323.03	324.04
4	Ethyl hydrogen sulfate	$C_2H_6O_4S$	124.99	126.00
5	beta-L-fucose	$C_6H_{12}O_5$	163.06	164.07
6	Adenosine5'-monophosphate	$C_{10}H_{14}N_5O_7P$	346.06	347.06
7	Guanidylic acid (guanosine monophosphate)	$C_{10}H_{14}N_5O_8P$	362.05	363.06
**8**	**Itaconic acid**	$C_5H_6O_4$	**129.02**	**130.03**
**9**	**Citric acid**	$C_6H_8O_7$	**191.02**	**192.03**
10	2,3-Dihydroxy-2,4-cyclopenta dien-1-one	$C_5H_4O_3$	111.01	112.02
11	Thr Glu His	$C_{15}H_{23}N_5O_7$	384.15	385.16
**12**	**Pyroglutamic acid**	$C_5H_7NO_3$	**128.04**	**129.04**
13	Pseudouridine	$C_9H_{12}N_2O_6$	243.06	244.07
14	Xanthine	$C_5H_4N_4O_2$	151.03	152.03
**15**	**Succinic acid**	$C_4H_6O_4$	**117.02**	**118.03**
16	L-Tyrosine	$C_9H_{11}NO_3$	180.07	181.07
**17**	**Vanillic acid 4-sulfate**	$C_8H_8O_7S$	**246.99**	**248.00**
18	L-Leucine	$C_6H_{13}NO_2$	130.09	131.09
19	2-Hydroxy-6-oxo-6-(2-hydrox yphenoxy)-hexa-2,4-dienoate	$C_{12}H_{10}O_6$	249.04	250.05
20	(±)-Glycerol 1,2-diacetate	$C_7H_{12}O_5$	175.06	176.07
**21**	**L-Phenylalanine**	$C_9H_{11}NO_2$	**164.07**	**165.08**
**22**	**Ptilosaponoside B**	$C_{27}H_{44}O_{11}S$	**575.25**	**576.26**

(a)

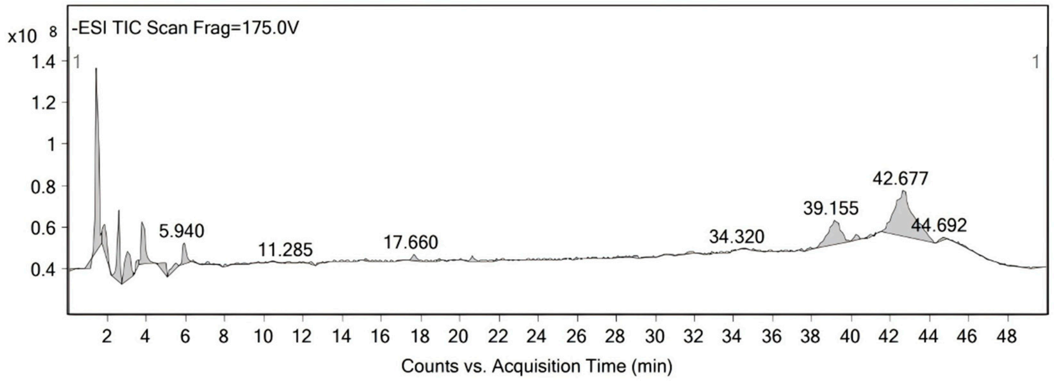

(b) Peak no. 2 Glyceric acid

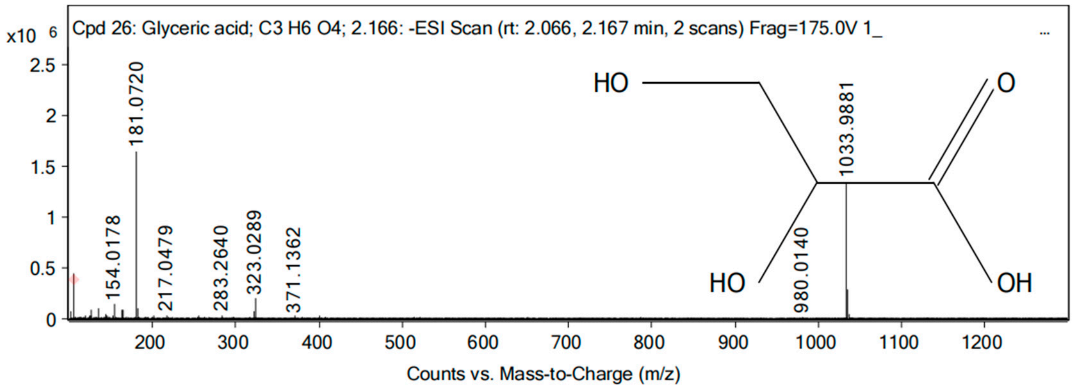

(c) Peak no. 8 Itaconic acid

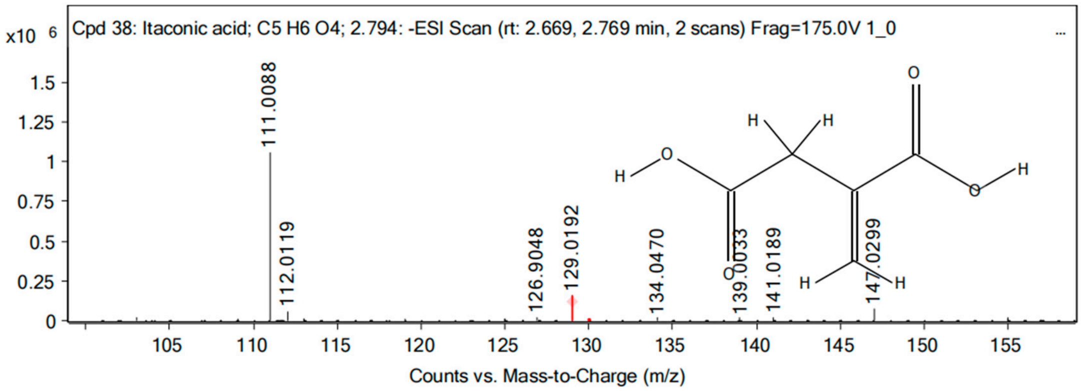

(d) Peak no. 9 Citric acid

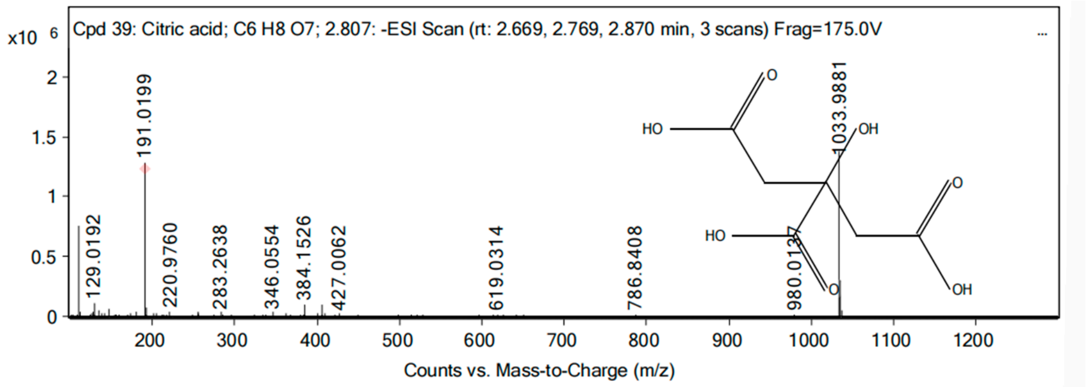

(e) Peak no. 12 Pyroglutamic acid

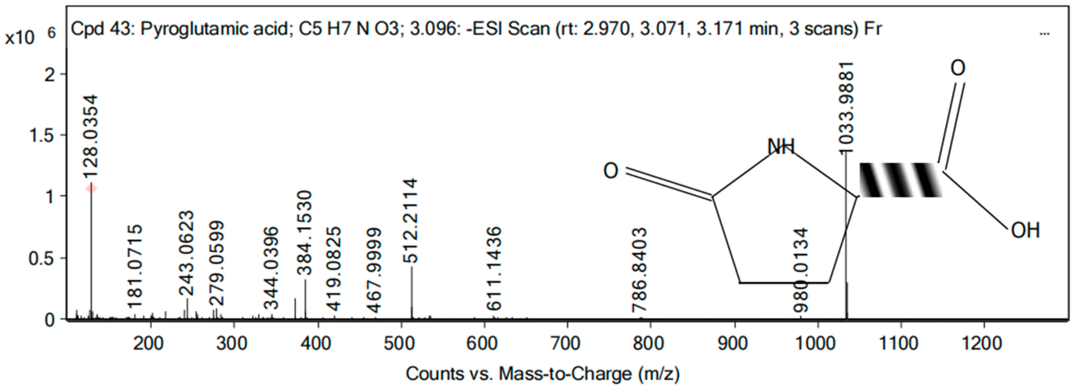

(f) Peak no. 15 Succinic acid

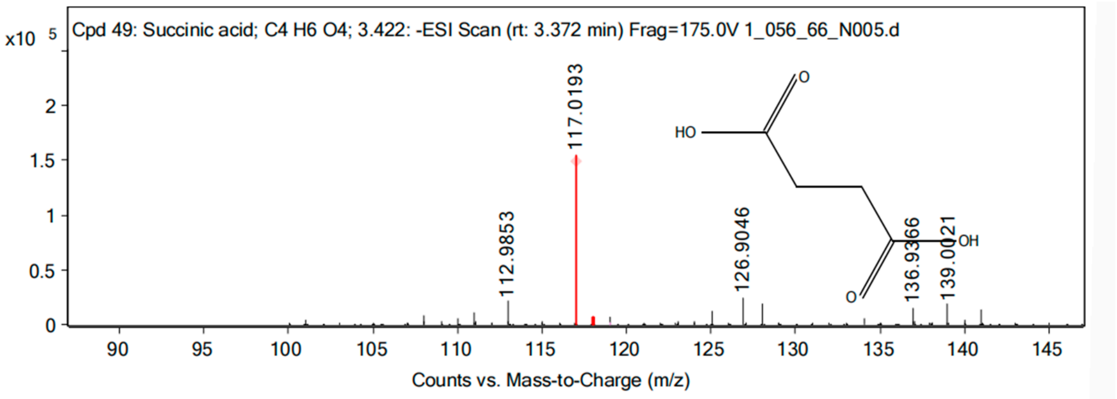

(g) Peak no. 17 Vanillic acid 4-sulfate

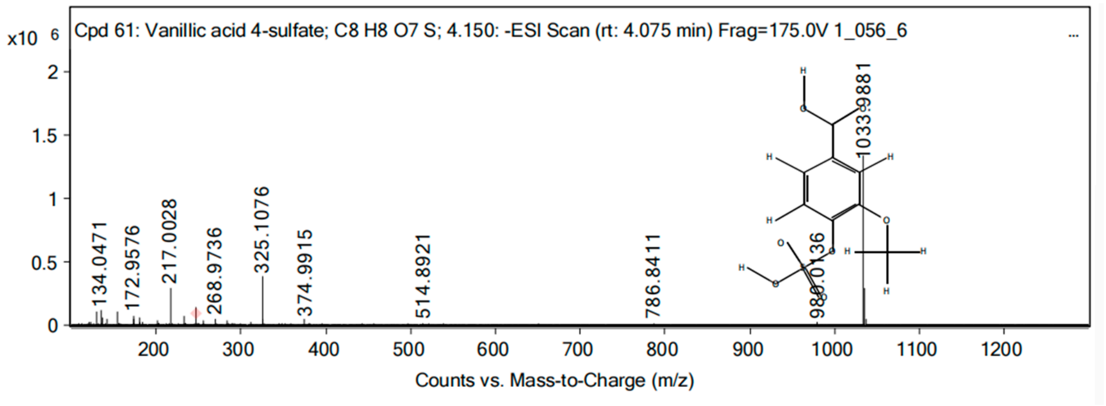

(h) Peak no. 21 L-Phenylalanine

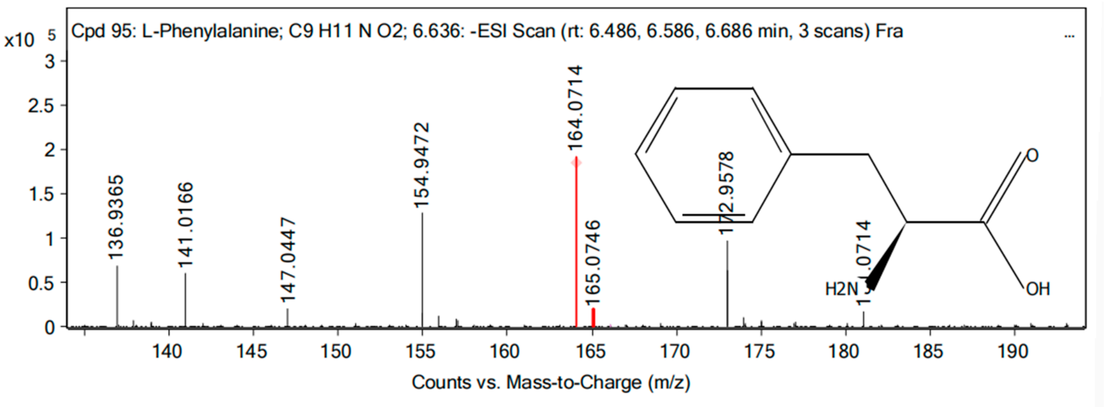

(i) Peak no. 22 Ptilosaponoside B

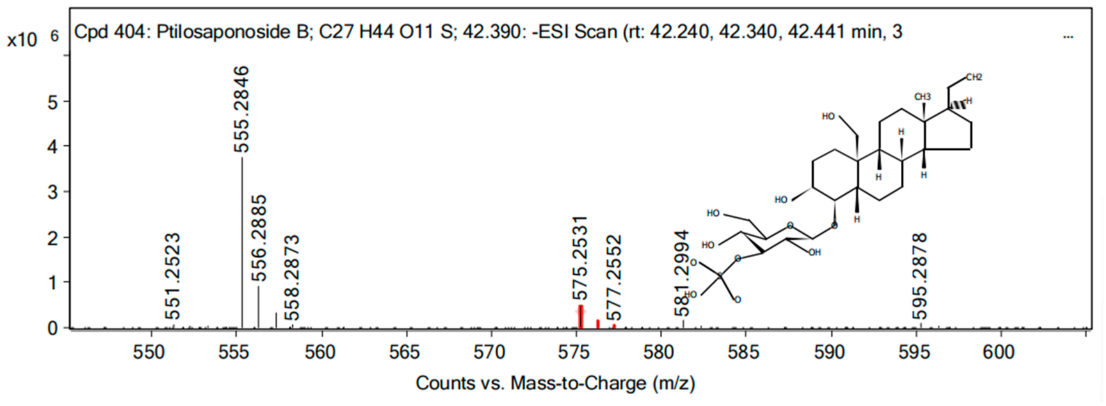

Figure S1 LC-MS chromatographic profiles of *H. fusiforme* extract (a) and potential functional compounds (b-i).

Table S3 LC-MS identification of components in *L. japonica* extract

Peak no.	ldentified compounds	Formula	m/z	Mass (g/mol)
1	Phosphatidyl glycerol	$C_6H_{15}O_8P$	245.04	246.05
2	D-Sorbitol	$C_6H_{14}O_6$	181.07	182.08
3	L-Glutamate	$C_5H_9NO_4$	146.05	147.05
4	Uridine monophosphate (UMP)	$C_9H_{13}N_2O_9P$	323.03	324.04
5	Guanidylic acid (guanosine monophosphate)	$C_{10}H_{14}N_5O_8P$	362.05	363.06
6	Adenosine5'-monophosphate	$C_{10}H_{14}N_5O_7P$	346.06	347.06
7	Deoxyadenosine monophosphate	$C_{10}H_{14}N_5O_6P$	330.06	331.07
**8**	**Uric acid**	$\mathbf{C_5H_4N_4O_3}$	**167.02**	**168.03**
9	Pyroglutamic acid	$C_5H_7NO_3$	128.04	129.04
10	Pseudouridine	$C_9H_{12}N_2O_6$	243.06	244.07
11	Xanthine	$C_5H_4N_4O_2$	151.03	152.03
**12**	**omega-Hydroxymoracin N**	$\mathbf{C_{19}H_{18}O_5}$	**325.11**	**326.12**
13	Adenosine	$C_{10}H_{13}N_5O_4$	266.09	267.10
14	Adenine	$C_5H_5N_5$	134.05	135.05
15	Guanosine	$C_{10}H_{13}N_5O_5$	282.08	283.09
16	3-Deoxyguanosine	$C_{10}H_{13}N_5O_4$	266.09	267.10
17	Telbivudine	$C_{10}H_{14}N_2O_5$	241.08	242.09
18	L-α-Hydroxyisovaleric acid	$C_5H_{10}O_3$	117.06	118.06
**19**	**Pyrocatechol**	$\mathbf{C_6H_6O_2}$	**109.03**	**110.04**
20	Glycerol tripropanoate	$C_{12}H_{20}O_6$	259.12	260.13
21	Methyl N-(a-methylbutyryl)glycine	$C_9H_{16}O_4$	187.10	188.10
**22**	**Cardamonin**	$\mathbf{C_{16}H_{14}O_4}$	**269.08**	**270.09**
**23**	**Myrsinone**	$\mathbf{C_{17}H_{26}O_4}$	**293.18**	**294.18**

(a)

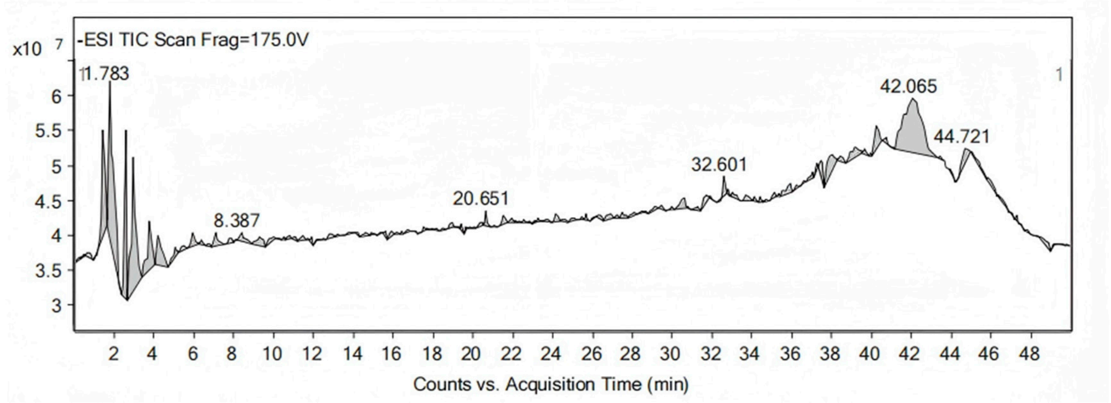

(b) Peak no.8 Uric acid

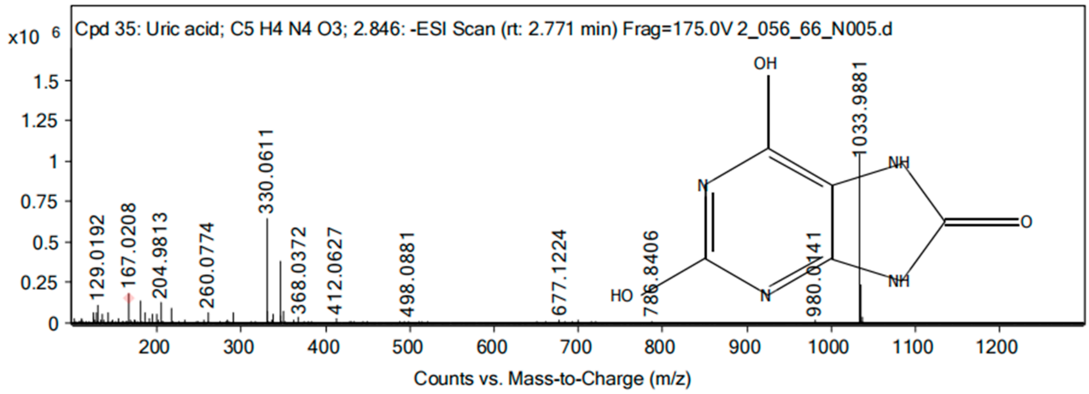

(c) Peak no.12 omega-Hydroxymoracin N

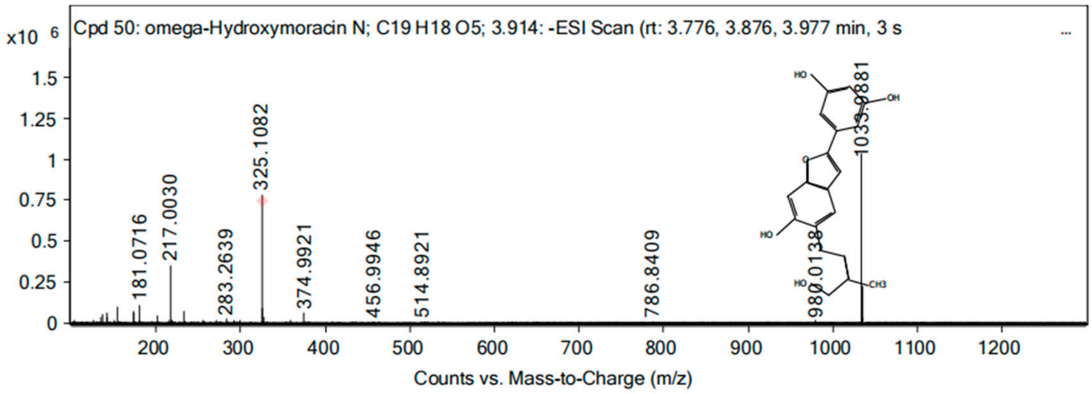

(d) Peak no.19 Pyrocatechol

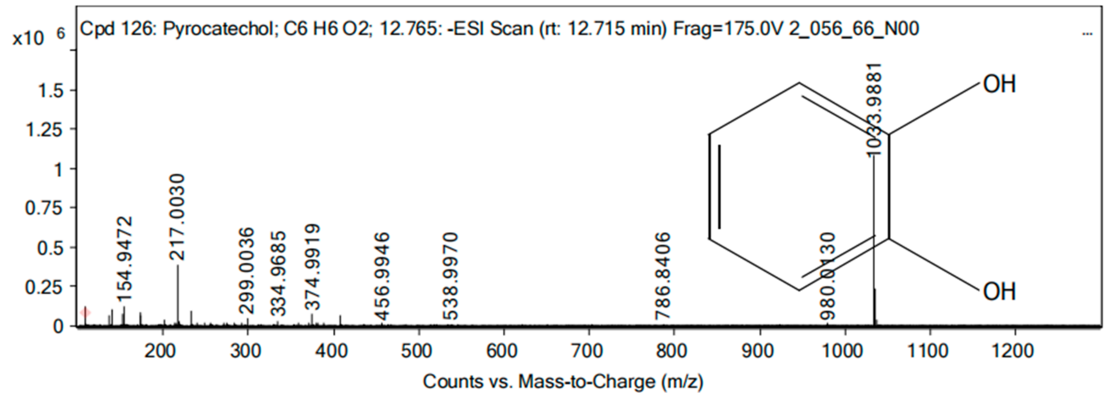

(e) Peak no.22 Cardamonin

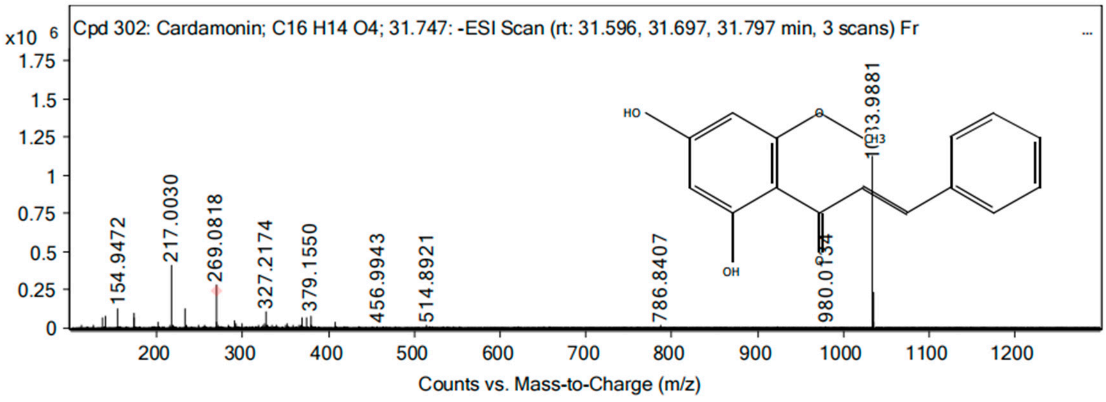

(f) Peak no.23 Myrsinone

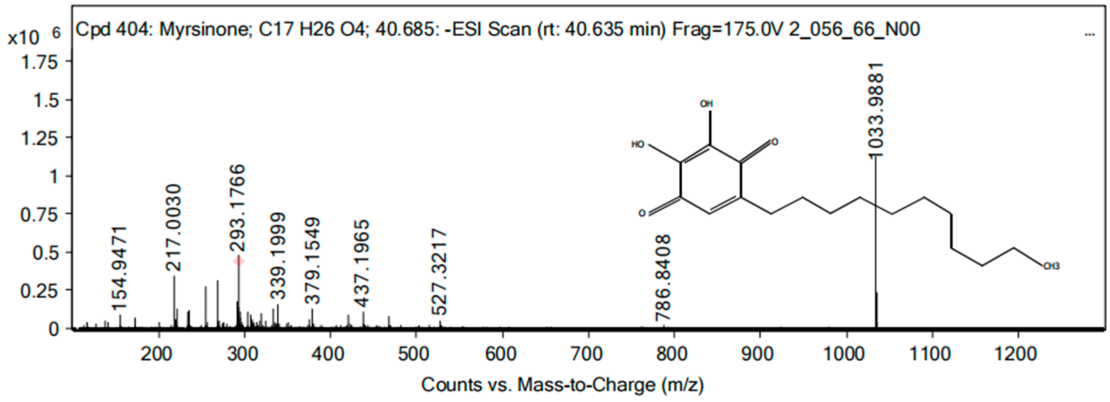

Figure S2 LC-MS chromatographic profiles of *L. japonica* extract (a) and potential functional compounds (b-f).
